# Effect of air pollution on the global burden of cardiovascular diseases and forecasting future trends of the related metrics: a systematic analysis from the Global Burden of Disease Study 2021

**DOI:** 10.3389/fmed.2024.1472996

**Published:** 2024-10-11

**Authors:** Qingsong Mao, Xiaoyi Zhu, Xinyi Zhang, Yuzhe Kong

**Affiliations:** ^1^Hepatobiliary Pancreatic Surgery, Banan Hospital Affiliated of Chongqing Medical University, Chongqing, China; ^2^Xiangya School of Medicine, Central South University, Changsha, China; ^3^College of Education, Wenzhou University, Wenzhou, China

**Keywords:** cardiovascular diseases, air pollution, mortality forecasting, epidemiology, disease burden

## Abstract

**Background:**

This study assesses the worldwide cardiovascular disease (CVD) burden attributed to air pollution, utilizing data from the Global Burden of Disease Study 2021.

**Methods:**

We explored the impact of air pollution on CVDs globally, regionally, and nationally, while considering correlations with age, gender, and socio-demographic index (SDI). A decomposition analysis was conducted to discern the contributions of aging, population growth, and epidemiological shifts to the changes in disability-adjusted life years (DALYs) from 1990 to 2021. Additionally, an ARIMA model was used to forecast the future CVD burden through 2050.

**Results:**

In 2021, air pollution was responsible for approximately 2.46 million deaths and 58.3 million disability-adjusted life years (DALYs) attributable to CVDs, with a discernible decrease over the period studied. The greatest impacts were observed in individuals aged 75–79 and over 80, particularly among males. The decomposition analysis indicated that shifts in epidemiology were the primary factors driving these changes. Future projections suggest potential increases in mortality and DALY rates in regions with low and high-middle SDI, alongside rising age-standardized death and mortality rates in high SDI areas.

**Conclusion:**

These findings underscore the urgency of implementing targeted CVD prevention and air pollution control strategies to mitigate the impact on public health.

## Graphical abstract

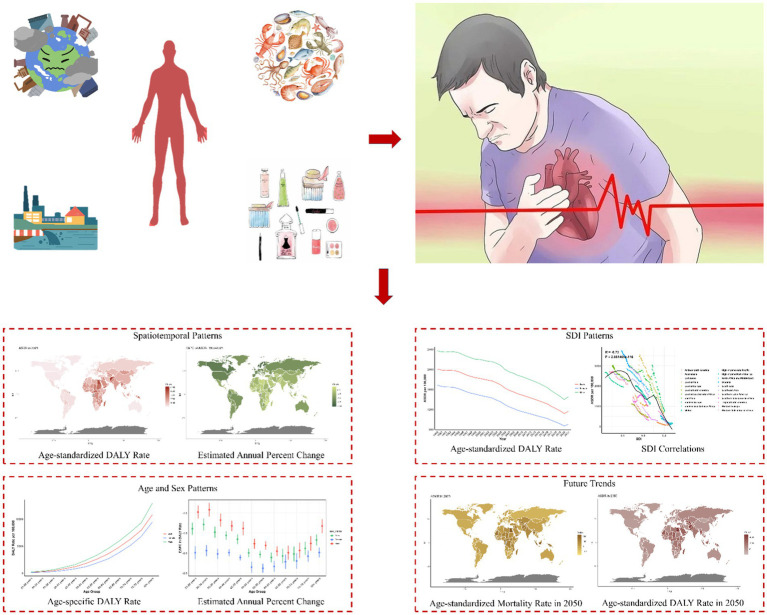

## 1 Introduction

Air pollution, a complex mixture of particulate matter and gases, is associated with approximately 8.8 million additional deaths annually. Nearly half of these deaths are linked to external environmental pollutants, while the remainder result from indoor pollution, substantially increasing the global health burden (1, 2). According to the World Health Organization (WHO) and the Global Burden of Disease Study, air pollution ranks as the fourth leading global cause of death and disease, just behind high blood pressure, tobacco use, and dietary risks (3). While the effects of air pollution on respiratory health are well-known, it is important to emphasize that cardiovascular diseases (CVDs) account for half of the deaths associated with air pollution (2). Additionally, solid evidence worldwide shows that approximately 20% of CVD deaths are related to exposure to air pollutants such as PM_2.5_, PM_10_, ozone (O_3_), and nitrogen dioxide (NO_2_) (4, 5). Beyond traditional risk factors, the impact of environmental factors like ambient air pollution is increasingly recognized as critical (6).

Extensive epidemiological studies demonstrate that air pollution aggravates cardiovascular risk factors including hyperlipidemia, hypertension, atherosclerotic changes, and diabetes, thus elevating the risk of cardiovascular conditions such as ischemic heart diseases, heart failure, and strokes (7–9). Several mechanisms, including oxidative stress induction, inflammation, disturbances in autonomic and neuroendocrine functions, increased vasoconstriction and coagulation, and particulate matter penetration into the bloodstream, are proposed to explain the connection between air pollution and CVDs (10, 11). Consequently, the link between air pollution and cardiovascular health has emerged as a critical issue in environmental and public health, necessitating comprehensive research to effectively identify and address this challenge.

In this research, we examined the impact of air pollution on the cardiovascular health burden, analyzing trends in mortality and DALYs across different age groups, genders, and socio-demographic indices from 1990 to 2021. We also projected future trends using the autoregressive integrated moving average (ARIMA) model, validated by multiple prior studies (12, 13). These results are intended to aid in decision-making regarding the prevention of cardiovascular diseases and the management of air pollution.

## 2 Method

### 2.1 Study data

Data for our study were obtained from the Global Burden of Disease Study (GBD) 2021, which is available at http://ghdx.healthdata.org/gbd-results-tool. The GBD 2021 provides a comprehensive assessment of 369 diseases and injuries, along with 87 risk factors across 204 countries spanning from 1990 to 2021 (2).

The specific methodologies employed to calculate the burden of cardiovascular diseases (CVDs) are elaborated in additional references [14, 15]. This summary describes the approach adopted in GBD 2021. Sources for CVD mortality data included vital registration systems, surveillance data, and verbal autopsies. This vital registration data was then refined to enhance accuracy, addressing data gaps and miscoding issues (16). These adjusted datasets were analyzed using the Cause of Death Ensemble model (CODEm), which generated estimates of CVD mortality segmented by location, year, age, and gender (15, 17). Furthermore, a comparative risk assessment was conducted to identify principal risk factors for CVDs. The population attributable fraction (PAF) was computed to quantify the contribution of air pollution to the CVD burden. Estimates for CVD mortality and disability-adjusted life years (DALYs) related to air pollution were derived by applying these specific PAFs to the mortality and DALY figures across different demographics (2).

DALYs serve as an inclusive metric of disease impact, combining years lost due to premature mortality (YLLs) and years lived with disability (YLDs). YLLs were calculated by multiplying the number of deaths from cardiovascular diseases in each age group by the remaining life expectancy for that age group. YLDs were determined by multiplying the prevalence of CVDs by the severity-adjusted disability weights (DWs) (16). The Socio-Demographic Index (SDI), a composite indicator ranging from 0 (worst) to 100 (best), was derived from three factors: the total fertility rate for individuals under 25 years old (TFU25), average educational attainment for those over 15 years old (EDU15+), and adjusted *per capita* income. Based on the SDI, the 204 countries and territories were classified into five categories: low SDI, low-middle SDI, middle SDI, high-middle SDI, and high SDI (16).

### 2.2 Risk factor estimate

In the Global Burden of Disease (GBD) study, the estimation of risk factors is guided by the comparative risk assessment (CRA) framework. This approach begins by determining the relative risk (RR) of specific health outcomes associated with exposure to risk factors, utilizing meta-regression and systematic reviews. Subsequent steps involve Bayesian statistical models, such as spatio-temporal Gaussian process regression (ST-GPR) and disease model meta-regression (DisMod-MR), to estimate the levels and distribution of exposure for each risk factor. Additionally, theoretical minimum risk exposure levels (TMREL) are established. These represent the exposure levels that would ideally minimize health risks. Based on these assessments, population attributable fractions (PAF) and summary exposure values (SEV) are calculated. These metrics indicate the potential changes in health outcomes that could result if exposures were reduced to TMREL. These calculations are essential for assessing the disease burden attributable to various risk factors.

### 2.3 Statistical analysis

Age-standardized rates (ASR) were utilized to normalize mortality and DALY rates across nations with varying age distributions and demographic profiles. A linear model was applied to the natural logarithm of these rates over time, formulated as *y* = *α* + *βx* + *ϵy*, where *xxx* represents the year, and *y* is the natural logarithm of the rate. The estimated annual percentage change (EAPC) was calculated as 100 × (*eβ* − 1)100, along with a 95% confidence interval (95% CI). An increase in ASR was identified when both the EAPC and the lower boundary of the 95% CI were positive. Conversely, a decrease was noted if the EAPC and the upper boundary of the 95% CI were negative. If neither condition was met, ASR was considered stable during the study period (18, 19).

The relationship between ASR and the socio-demographic index (SDI) was examined using a Gaussian process regression framework with Loess smoothing and assessed through Spearman rank order correlation tests (18, 20). A decomposition analysis quantified the impacts of aging populations, population growth, and epidemiological shifts on overall DALY changes from 1990 to 2021, with methodologies detailed in earlier publications (21).

Additionally, the ARIMA model was employed to evaluate the influence of air pollution on CVD trends and forecast global, regional, and national trends from 2020 to 2050. Known formally as the “integral moving average autoregressive model,” the ARIMA model integrates differential, integral, moving average, and autoregressive components. In the ARIMA model (*p*, *d*, *q*), “AR” signifies autoregressive, with *ppp* denoting the number of autoregressive terms; “MA” represents the moving average component, with *qqq* as the number of moving average terms; and *ddd* refers to the number of differencing steps to achieve stationarity (22). Model selection was optimized using the Akaike information criterion (AIC) and the Bayesian information criterion (BIC).

A 95% uncertainty interval (UI) was calculated for all measurements. Rates were expressed per 100,000 individuals. Data management, analysis, and visualization were performed using R software version 4.3.2.

## 3 Result

### 3.1 Spatiotemporal patterns of CVD attributable to air pollution

In 2021, CVD led to about 19.41 million deaths and 428.33 million disability-adjusted life years (DALYs). Among them, air pollution led to approximately 2.46 million deaths and 58.30 million DALYs due to cardiovascular diseases (CVDs), with an age-standardized mortality rate (ASMR) of 53.62 (95% UI, 42.70–64.57) and an age-standardized DALY rate (ASDR) of 1161.77 (95% UI, 939.61–1380.37) per 100,000 population. Over the last thirty years, the CVD burden from air pollution has shown a significant decline (Tables 1, 2; Supplementary Table S1).

**Table 1:** Global and regional deaths and DALYs of CVDs attributable to air pollution in 1990 and 2021 in 27 global regions and different genders.

Location	Deaths number in 1990	Deaths number in 2021	ASMR in 2021	DALY number in 1990	DALY number in 2021	ASDR in 2021
Global	1695327.4670 (1977170.5901, 1427078.5915)	2468592.2959 (2971801.8642, 1953863.3588)	53.6174 (64.5662, 42.6994)	43362305.0129 (50272581.2571, 36668710.4699)	58299045.0825 (69763497.3333, 46981815.3298)	1161.7722 (1380.3722, 939.6053)
Gender						
Male	1695327.4670 (19771705901, 1427078.5915)	2468592.2959 (2971801.8642, 1953863.3588)	66.2955 (80.0582, 52.1721)	43362305.0129 (50272581.2571, 36668710.4699)	58299045.0825 (69763497.3333, 46981815.3298)	1452.7201 (1738.2851, 1168.4189)
Female	1630076.9204 (1927676.1020, 1357969.1191)	2013903.9322 (2422658.2196, 1592567.0145)	43.2348 (51.9769, 34.2335)	35510624.1810 (41686369.9278, 29789730.0616)	41338792.0101 (49169185.9168, 33240171.4369)	900.6461 (1071.2889, 724.6623)
Region						
East Asia	995635.1048 (1184133.8618, 820235.5094)	1516109.2462 (1921799.6028, 1159124.9502)	79.4032 (100.0644, 60.9000)	24088329.7918 (28642768.5871, 19747638.0085)	29566224.4247 (37600098.9963, 22695124.2268)	1432.5120 (1818.5155, 1105.6533)
Southeast Asia	292409.4259 (341167.7601, 239156.6766)	414478.2168 (524871.4679, 310254.2424)	72.8560 (91.8257, 54.3598)	7702975.3625 (9009587.4587, 6351206.5927)	10202762.5808 (13097329.4276, 7643198.8456)	1566.8157 (1993.6971, 1173.1989)
Oceania	3929.2258 (5022.2345, 3006.3087)	8135.5507 (10428.4797, 6025.1548)	128.1500 (161.9897, 96.6313)	115694.5127 (149599.5885, 87715.1994)	235289.2364 (306964.9383, 172427.2916)	2941.5803 (3758.2603, 2174.6057)
Central Asia	54552.0623 (76539.5652, 35526.7865)	65599.1344 (82696.3388, 49613.5264)	98.4258 (124.2898, 74.0839)	1195900.0039 (1687856.4669, 778351.2762)	1431146.6198 (1794120.5349, 1078832.1102)	1872.0913 (2343.7756, 1413.9035)
Central Europe	177291.2836 (239604.7043, 114951.4549)	90168.6410 (121110.2728, 67837.2020)	37.9224 (50.9129, 28.5961)	3572446.0165 (4806187.2707, 2325299.1638)	1522029.8266 (2041946.1803, 1155972.7451)	676.7089 (907.4438, 514.0276)
Eastern Europe	310543.6613 (458160.8375, 164812.6808)	153473.3540 (229652.7298, 96088.6386)	42.9521 (64.2367, 26.9005)	6153494.9567 (9048930.7816, 3285095.5912)	2797506.8040 (4112752.6421, 1770610.3146)	802.1190 (1178.6264, 508.1216)
High-income Asia Pacific	38250.0681 (72961.7274, 10974.1797)	41800.6376 (60312.0967, 24737.6891)	7.0048 (9.9909, 4.3139)	808578.9546 (1543555.9978, 240764.7608)	710535.7491 (1007262.9837, 437006.0259)	153.6023 (214.8820, 95.3220)
Australasia	2729.0431 (7552.4241, 94.7367)	2791.2600 (4198.1991, 1580.7685)	4.5055 (6.7547, 2.5404)	50811.4323 (139619.9939, 1748.5420)	44476.0194 (66198.6791, 25642.4317)	80.3321 (119.0927, 46.3121)
Western Europe	247398.3436 (397173.1254, 122625.0513)	74452.9037 (100642.9088, 50578.4214)	6.3631 (8.5546, 4.3541)	4361300.3912 (6948664.6399, 2168340.0610)	1144443.1203 (1543429.8090, 802248.2437)	114.8251 (153.7857, 80.5935)
Southern Latin America	21634.4777 (32705.1743, 11850.3467)	11828.1789 (17569.9612, 6841.0366)	13.0890 (19.4295, 7.5780)	454923.7033 (691979.6497, 247450.9039)	233587.5870 (345270.5634, 135950.4403)	268.4134 (396.6459, 156.2518)
High-income North America	99492.2929 (175695.9489, 40359.6178)	31507.1887 (51217.4270, 15009.8341)	4.4593 (7.2487, 2.1248)	1843521.2401 (3222999.0891, 753865.0119)	575238.1857 (930447.9526, 273918.2634)	89.2999 (143.9604, 42.4338)
Caribbean	18296.4156 (25448.2459, 12855.5225)	24251.5860 (32896.1005, 16545.4105)	44.7410 (60.6390, 30.5579)	420823.9449 (572012.7382, 305366.5731)	550668.7510 (743129.9627, 381954.1997)	1027.5418 (1385.4111, 712.8068)
Andean Latin America	13175.8205 (15939.8672, 10547.5143)	12758.3159 (17546.6813, 8973.7710)	22.4437 (30.8327, 15.7920)	312233.6889 (377692.6302, 250627.4808)	274209.7435 (378920.8468, 193125.8763)	461.8767 (637.9337, 325.3280)
Central Latin America	40212.7970 (53052.4395, 28048.6728)	54983.4862 (75020.3826, 38196.7440)	23.0967 (31.5161, 16.0590)	919916.7348 (1211183.4959, 639052.7796)	1135777.3836 (1559780.7654, 791892.2982)	457.1114 (626.5176, 318.6461)
Tropical Latin America	48310.5756 (70390.5280, 28728.2208)	34779.9324 (50098.6078, 20256.0361)	13.8874 (20.0204, 8.0993)	1193491.5822 (1745901.5364, 697242.6729)	798762.5859 (1146605.8902, 469850.4417)	309.6700 (444.2645, 182.1120)
North Africa and Middle East	191712.9995 (226694.2273, 155481.6693)	339831.9837 (409989.3193, 271225.7511)	89.0225 (106.7349, 70.8306)	4774495.4770 (5680593.8070, 3857682.7204)	8132960.8611 (9907073.5884, 6451588.1781)	1798.5771 (2174.0080, 1432.8545)
South Asia	563019.8302 (651144.2453, 474660.4883)	1250304.4615 (1462892.3775, 1045358.4616)	94.4647 (110.4090, 78.8850)	15603270.2799 (18064917.9081, 13253674.7665)	31263672.8437 (36643862.9574, 26098304.4684)	2094.1080 (2452.6221, 1747.3016)
Central Sub-Saharan Africa	24284.0787 (30168.2275, 19401.9787)	45323.0476 (58644.6815, 33622.3074)	110.5971 (141.6104, 82.5695)	647568.1295 (808427.4001, 511470.4147)	1182638.7094 (1532681.5079, 889409.9251)	2274.3773 (2916.8406, 1710.0685)
Eastern Sub-Saharan Africa	74408.0979 (88017.8048, 61939.9862)	125700.9343 (148776.4502, 103606.9149)	91.2503 (108.3014, 75.3993)	2003936.8682 (2383524.5962, 1674514.5775)	3300546.9000 (3914092.0739, 2707095.3723)	1956.3214 (2314.0052, 1606.0484)
Southern Sub-Saharan Africa	13193.8831 (16261.9577, 10198.8335)	23178.0975 (28655.6990, 17586.1958)	47.8778 (59.2397, 35.9981)	338946.6340 (415012.3883, 267825.3248)	567046.6232 (705509.3680, 433808.4655)	1001.0289 (1241.6501, 761.5670)
Western Sub-Saharan Africa	94924.9002 (111889.2557, 78007.9258)	161040.0710 (193364.8423, 132163.3701)	104.0693 (123.5385, 85.2558)	2310269.4889 (2708637.4995, 1894797.4127)	3968312.5373 (4808350.4749, 3245544.7257)	2093.3947 (2506.8769, 1720.4055)
SDI						
High-middle SDI	940336.2440 (1159157.5696, 729244.2038)	922495.5999 (1176292.3187, 715704.0364)	47.7537 (60.8469, 37.0495)	20436983.5742 (24937334.2661, 16099108.2518)	17518215.6278 (22039734.3635, 13707867.1341)	900.2615 (1131.6321, 704.3709)
High SDI	446201.4186 (614797.8107, 307147.5465)	237576.9488 (310056.3864, 172797.2332)	10.1980 (13.2477, 7.4142)	8531364.7135 (11698914.5076, 5950760.1991)	4419388.9444 (5675549.5283, 3274373.5386)	221.0099 (281.7897, 165.4033)
Low-middle SDI	648976.4581 (742318.5502, 556313.4875)	1226912.9175 (1425316.9868, 1013901.1946)	95.6295 (110.9862, 79.1204)	17329837.8986 (19792485.6239, 14827104.6637)	30380359.1914 (35460141.3211, 25206113.8128)	2097.5866 (2440.9952, 1745.3074)
Low SDI	245189.5103 (283760.9366, 206886.3566)	444246.5011 (519262.7157, 374845.7863)	105.8942 (123.7471, 89.1028)	6540402.8771 (7683839.9868, 5548105.3090)	11425503.8763 (13300421.1318, 9588140.9866)	2256.3035 (2621.3672, 1901.4367)
Middle SDI	1040204.5101 (1213776.2974, 883408.7472)	1647692.7766 (2040082.4543, 1290448.7984)	69.2519 (85.9377, 54.2699)	25936668.0362 (30162567.1744, 22048394.1061)	35817251.1043 (44013141.4681, 28380713.0044)	1373.5525 (1688.9324, 1083.3614)

**Table 2:** Global and regional deaths and DALYs of CVDs in 1990 and 2021 in 27 global regions and different genders.

Location	Deaths number in 1990	Deaths number in 2021	ASMR in 2021	DALY number in 1990	DALY number in 2021	ASDR in 2021
Global	12330009.3008 (12787471.5911, 11626404.9865)	19414853.0875 (20668512.4937, 17775807.4640)	358.1215 (372.6290, 333.7411)	297507308.4325 (309345618.5144, 284600934.8335)	428327412.2838 (453711663.4307, 403683550.4114)	7550.1684 (7861.9927, 7181.4625)
Region						
East Asia	2576813.0423 (2859920.2498, 2280964.4437)	5231174.2615 (6056337.1435, 4452990.2327)	403.3261 (445.8899, 358.3746)	65291854.4059 (72391953.4755, 58394399.0585)	103566223.2648 (119867679.8848, 88067786.6165)	8014.8092 (8844.4747, 7134.0810)
Southeast Asia	779349.4010 (847499.5624, 707641.0304)	1721548.8969 (1858463.1825, 1556629.3188)	355.4948 (388.6949, 318.9347)	21924178.7091 (23640597.2082, 20172071.1971)	43674125.2338 (47589095.9866, 39509277.9960)	8110.3431 (8770.8906, 7393.6121)
Oceania	11289.6878 (13443.0606, 9434.8786)	24586.7000 (28674.9237, 20667.7626)	449.4996 (525.9145, 384.6594)	369579.8689 (444821.4230, 303941.5262)	776725.4554 (908794.9210, 653399.8410)	10865.7674 (12854.3455, 9179.0339)
Central Asia	214254.9701 (220440.5444, 203736.0545)	293313.0305 (320330.0544, 266419.5501)	512.8786 (529.7830, 484.1572)	4942615.0157 (5078554.9336, 4785143.0742)	6687963.5111 (7313824.8914, 6082677.6937)	10668.1128 (10979.0799, 10266.4394)
Central Europe	723472.2920 (737744.3438, 695750.3565)	682173.6929 (724975.7907, 621708.1279)	542.7664 (555.7813, 516.1485)	14920440.2024 (15224614.3845, 14534005.3153)	11848309.6931 (12583230.4713, 11046369.9976)	10469.0891 (10700.4813, 10134.1201)
Eastern Europe	1396706.8362 (1422674.7668, 1336338.0723)	1490605.8459 (1620588.7552, 1348328.8061)	570.0931 (583.4916, 540.1170)	28872233.1837 (29398196.5216, 28062119.9211)	29076844.1886 (31492927.0337, 26856664.0640)	10931.9273 (11150.4104, 10552.2126)
High-income Asia Pacific	358927.9579 (375092.2397, 325959.9957)	455989.5758 (507729.1746, 363780.5726)	204.0999 (214.9380, 182.1473)	7544628.9715 (7897779.5337, 7086522.6476)	7666056.7918 (8355265.4717, 6619060.7738)	3963.7126 (4154.8775, 3694.1503)
Australasia	62953.6543 (65359.0892, 58248.7203)	58242.9703 (62867.0376, 49260.4423)	280.0207 (291.8992, 256.7591)	1212270.0067 (1256133.9364, 1147288.3956)	1015701.6930 (1095079.2399, 911364.1918)	5257.7478 (5456.7154, 4963.5399)
Western Europe	1665937.2221 (1730874.5805, 1523643.7827)	1262954.4286 (1367243.8049, 1058669.3475)	280.6833 (292.1707, 256.2185)	30161814.8246 (31253667.4484, 28345011.9180)	20513501.7672 (22129350.3988, 18067114.0050)	5250.4181 (5440.5721, 4940.3821)
Southern Latin America	133794.2903 (137286.7594, 127096.4100)	124511.5743 (131073.1291, 113757.3894)	315.6093 (324.9318, 297.1318)	2945851.3022 (3026762.3772, 2855268.1607)	2531298.4115 (2679611.4157, 2378424.0031)	6512.5276 (6699.4990, 6287.0104)
High-income North America	943627.0238 (988434.4061, 849456.5935)	981389.5367 (1048167.4518, 850395.5490)	260.3662 (272.6217, 234.7756)	18479329.5920 (19208197.5291, 17298841.8786)	19199990.7353 (20356283.8331, 17487538.1837)	5317.3019 (5520.3279, 4998.1887)
Caribbean	82020.0013 (84989.2807, 78133.0041)	124209.4431 (138459.8294, 110466.5551)	341.9506 (354.2058, 324.2097)	1961558.1233 (2047308.1442, 1872099.6810)	2812678.0958 (3177235.1090, 2485295.6760)	7393.0156 (7707.9326, 7063.2077)
Andean Latin America	37598.8584 (40794.4587, 34861.4944)	65684.2536 (77453.3339, 56153.1207)	196.8385 (212.8347, 182.5495)	1027370.3886 (1122150.9886, 952090.8741)	1559288.9482 (1816182.3757, 1343402.4403)	4464.7388 (4866.5559, 4136.2482)
Central Latin America	165207.3957 (168745.8954, 157753.6061)	389476.7679 (430749.4793, 347067.9626)	230.6379 (236.6198, 217.5443)	4140616.9563 (4258109.8328, 4012380.8781)	8547908.8009 (9503224.0946, 7750213.8514)	4768.8819 (4908.9838, 4583.6024)
Tropical Latin America	263566.1701 (270019.0280, 250151.8200)	385370.4228 (405260.4335, 351506.5280)	331.6954 (343.0030, 307.9601)	7013551.7840 (7197413.5083, 6776423.1719)	9311288.4962 (9741721.9071, 8763364.2566)	7379.5378 (7597.2616, 7055.8757)
North Africa and Middle East	750006.0342 (796221.3285, 698870.8066)	1354625.6239 (1496618.1344, 1210244.2987)	529.2313 (560.6171, 486.4433)	21342633.6285 (22683423.7468, 20071414.4726)	32979048.0349 (36634917.6372, 29306589.0025)	11421.7951 (12113.6948, 10721.0615)
South Asia	1554654.6927 (1674221.6693, 1402490.3612)	3666331.0215 (3957645.1533, 3381321.5306)	302.4687 (326.8040, 271.0688)	47373764.7790 (50779957.1619, 43286333.8555)	95358824.8320 (103033020.9081, 88177000.1955)	7379.8527 (7927.0677, 6697.1391)
Central Sub-Saharan Africa	73256.3412 (84984.4934, 61934.2320)	149150.9405 (183435.7965, 118949.9182)	410.9305 (468.3326, 351.6018)	2250982.6949 (2610691.4511, 1884518.2975)	4227600.1489 (5195234.5815, 3368631.7169)	9133.9576 (10496.7405, 7864.2023)
Eastern Sub-Saharan Africa	210769.1924 (228569.9272, 194575.9219)	356705.9634 (398573.9455, 321396.5816)	330.3295 (356.8587, 302.7167)	6439236.1045 (7027602.2787, 5899708.8856)	10431008.8497 (11625438.0897, 9366792.2742)	7631.4657 (8265.7185, 7036.8033)
Southern Sub-Saharan Africa	58586.2196 (63696.1104, 52618.4025)	130763.4896 (139193.6204, 121926.3735)	245.7243 (269.3870, 217.5461)	1652825.9183 (1773301.5238, 1523913.2459)	3353216.3171 (3607692.6148, 3142540.3469)	5652.4959 (6108.9394, 5145.1601)
Western Sub-Saharan Africa	267218.0175 (296708.8591, 238738.2933)	466044.6479 (528620.2882, 403942.6564)	354.2536 (393.3463, 315.8998)	7639971.9725 (8425312.7644, 6798257.4829)	13189809.0145 (15117888.6325, 11149879.4229)	7842.4565 (8690.1385, 7063.8065)
SDI						
High-middle SDI	3623409.5643 (3766722.9311, 3426829.2003)	5064431.4681 (5476622.4399, 4543851.2539)	433.5722 (451.9934, 403.5012)	79552322.2560 (82962332.1054, 75496470.0808)	97878833.4741 (105738655.6716, 89801819.4608)	8451.9075 (8819.1507, 8001.6279)
High SDI	3068129.4424 (3182090.3689, 2812106.7070)	2915728.7671 (3128618.0151, 2508058.9734)	279.2932 (290.5016, 255.0388)	59840124.1655 (62029530.3406, 56518437.7455)	53384546.7937 (57086143.3677, 48351709.1539)	5494.1853 (5699.9773, 5185.4587)
Low-middle SDI	1856725.4157 (1963813.3798, 1731444.8314)	3824731.3598 (4079680.7172, 3556182.8735)	345.3859 (367.3506, 319.2504)	55008142.0762 (58047504.3087, 51439492.2139)	98787647.8476 (105635894.9544, 92030849.8732)	8149.9047 (8594.9050, 7607.6605)
Low SDI	688215.5914 (746087.4835, 631318.5692)	1244981.6526 (1364117.4472, 1131577.4158)	352.1112 (380.9192, 319.5988)	20968132.6054 (22956100.9150, 19071303.4865)	35335445.4576 (39118035.3762, 31846845.2577)	8236.2574 (8920.5482, 7566.4863)
Middle SDI	3075367.7597 (3278008.5207, 2863825.1519)	6344628.4227 (6879029.9542, 5787321.9488)	368.9258 (393.4526, 339.3824)	81736385.6887 (87079930.4453, 76736379.6902)	142519012.5713 (152962109.3008, 132081340.0390)	7843.6036 (8338.5158, 7348.2878)

**Figure 1:**
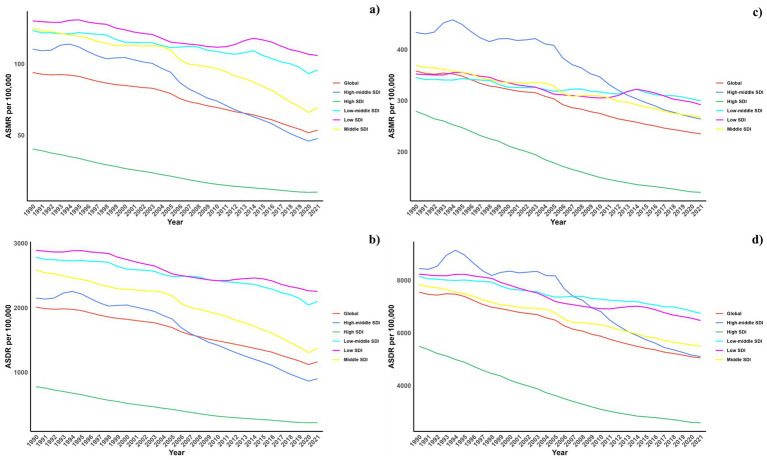
Temporal trends of ASMR and ASDR of CVDs attributable to air pollution (a,b) and total CVD burden (c,d) from 1990 to 2021 in different SDI regions.

Regarding socio-demographic index (SDI) regions, higher SDI regions experienced a notably lower CVD burden due to air pollution. In contrast, the low, low-middle, and middle SDI regions observed substantial increases in CVD burdens linked to air pollution. All SDI regions recorded slight reductions in both ASMR and ASDR due to air pollution. This spatiotemporal pattern of CVD burden was aligned with this (Table 1; Supplementary Table S1; Table 2, and Figure 1).

Regionally, East Asia, Southeast Asia, and East Asia reported the highest CVD burdens from air pollution, marked by the greatest numbers of deaths and DALYs. Conversely, Oceania, Central Sub-Saharan Africa, and Western Sub-Saharan Africa recorded the highest rates of ASDR and ASMR. While increases in ASMR due to air pollution were noted across all regions, decreases were seen in the European and American regions, except in Central Latin America. Similarly, ASDR trends generally increased, except in high-income Asia Pacific and Australasia where decreases were observed (Tables 1, 2). This spatiotemporal pattern of CVD burden was aligned with this.

**Figure 2:**
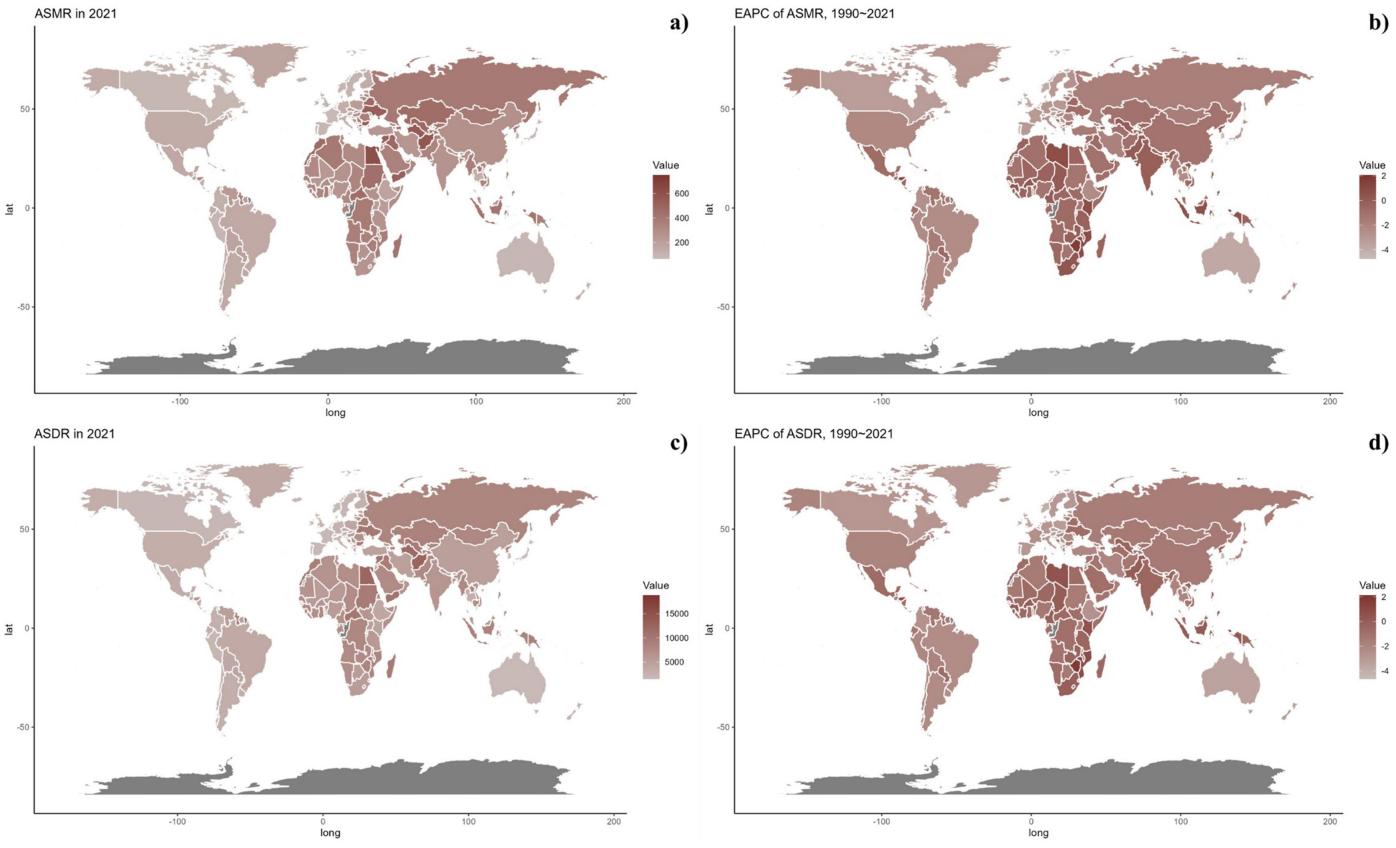
Global distribution of ASMR (a) and ASDR (c) of CVDs for both sexes in 2021 in 204 countries and territories. EAPC of ASMR (b) and ASDR (d) of CVDs from 1990 to 2021 in 204 countries and territories.

**Figure 3:**
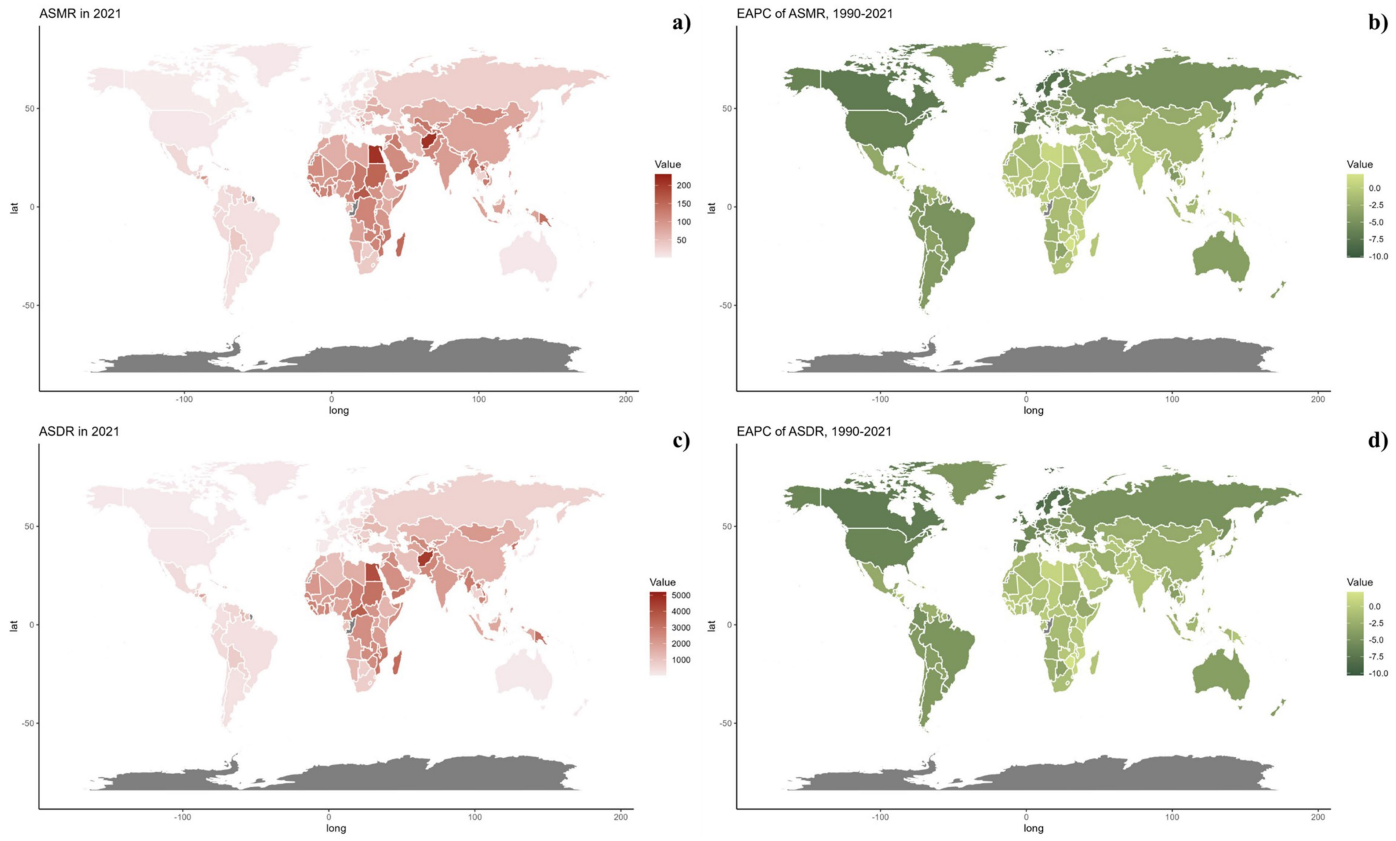
Global distribution of ASMR (a) and ASDR (c) of CVDs attributable to air pollution for both sexes in 2021 in 204 countries and territories. EAPC of ASMR (b) and ASDR (d) of CVDs attributable to air pollution from 1990 to 2021 in 204 countries and territories.

Nationally, in 2021, the ASDR of CVDs related to air pollution varied widely worldwide, with the highest rates noted in countries across North Africa, the Middle East, and Central Asia (Figure 2). From 1990 to 2019, most nations saw a decline in ASDR linked to air pollution, with the exception of 15 countries including Honduras [Estimate (95% UI): 0.1202 (0.3439, −0.1029)], Libya [Estimate (95% UI): 0.7887 (1.1774, 0.4014)], United Arab Emirates [Estimate (95% UI): −0.7593 (−0.2459, −1.2701)], Kenya [Estimate (95% UI): 0.4531 (0.6890, 0.2178)], Mozambique [Estimate (95% UI): 0.9241 (1.1264, 0.7222)], Lesotho [Estimate (95% UI): 2.1309 (2.6863, 1.5785)], Zimbabwe [Estimate (95% UI): 1.9544 (2.5467, 1.3656)], Burkina Faso [Estimate (95% UI): 0.0403 (0.1177, −0.0370)], Cameroon [Estimate (95% UI): 0.0070 (0.4280, −0.4121)], Chad [Estimate (95% UI): 0.1475 (0.3563, −0.0608)], Gambia [Estimate (95% UI): 0.1015 (0.2334, −0.0303)], Guinea [Estimate (95% UI): 0.3354 (0.4689, 0.2021)], Sierra Leone [Estimate (95% UI): 0.0179 (0.2127, −0.1765)], Northern Mariana Islands [Estimate (95% UI): 0.1044 (0.7569, −0.5440)], and Palau [Estimate (95% UI): 0.0144 (0.8653, −0.8294)]. The spatial and temporal trends of ASMR paralleled those of ASDR (Table 1; Supplementary Table S1, and Figures 2, 3). This spatiotemporal pattern of CVD burden was aligned with this.

### 3.2 Age and sex patterns

**Figure 4:**
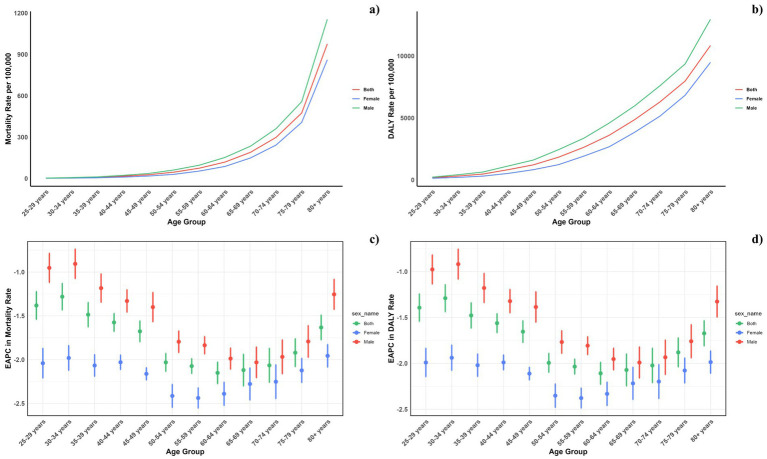
Age-specific rates of global deaths (a) and DALYs (b) of CVDs attributable to air pollution, by sex, in 2021 and the corresponding EAPC of global deaths (c) and DALYs (d) from 1990 to 2021.

**Figure 5:**
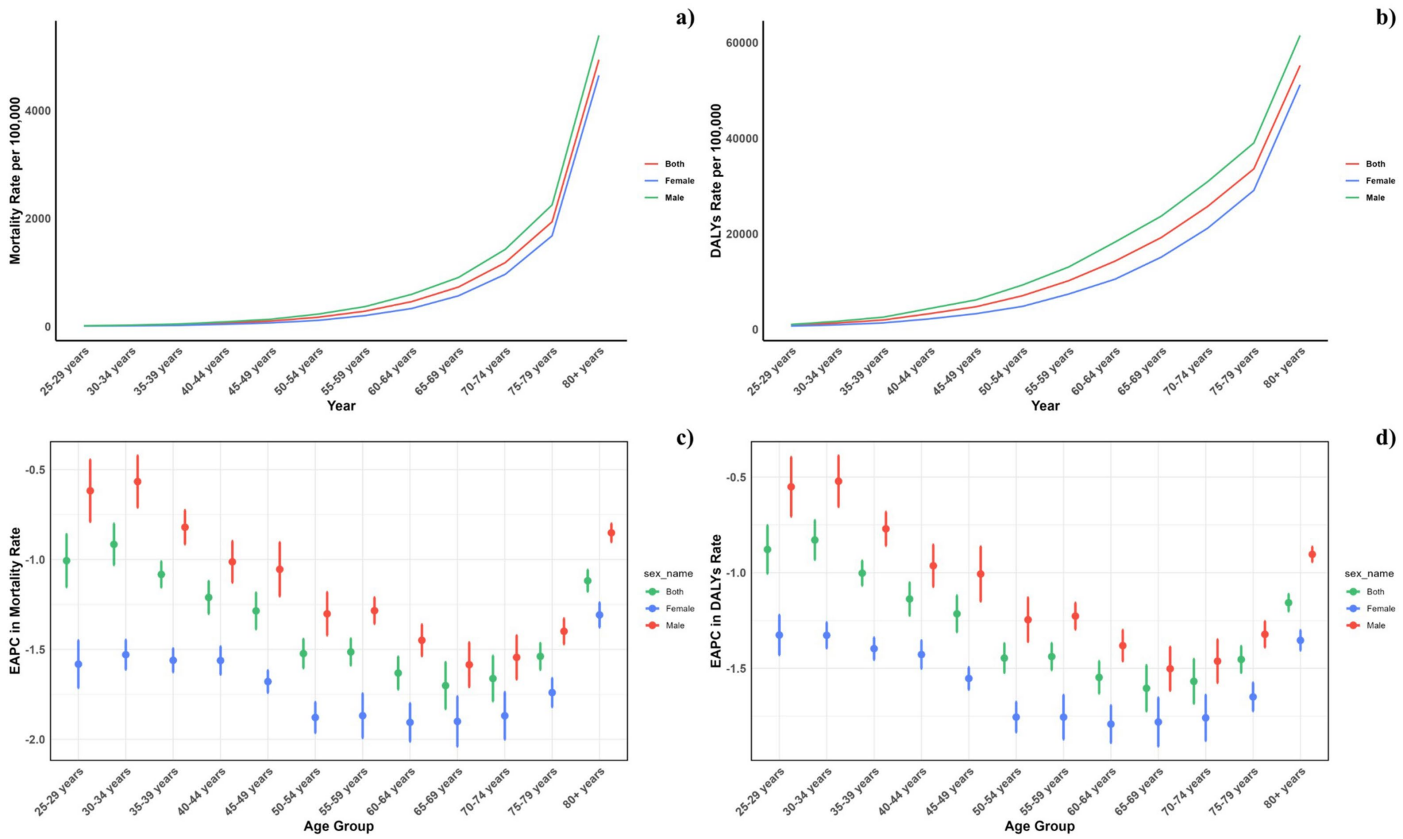
Age-specific rates of global deaths (a) and DALYs (b) of CVDs, by sex, in 2021 and the corresponding EAPC of global deaths (c) and DALYs (d) from 1990 to 2021.

Figure 4 displays the age-specific global mortality and DALY rates for CVDs in 2021, along with their changes from 1990 to 2021. These rates demonstrate a J-shaped distribution, showing a rise in mortality and DALY rates among individuals under 75, with a notable escalation in the 75–79 and 80+ age brackets. In all age categories, males consistently exhibited higher mortality rates due to air pollution compared to females. Likewise, the DALY rates linked to air pollution were greater for all age groups. Similarly, it can be also found that for the total CVD burden, males also suffered more (Figure 5).

**Figure 6:**
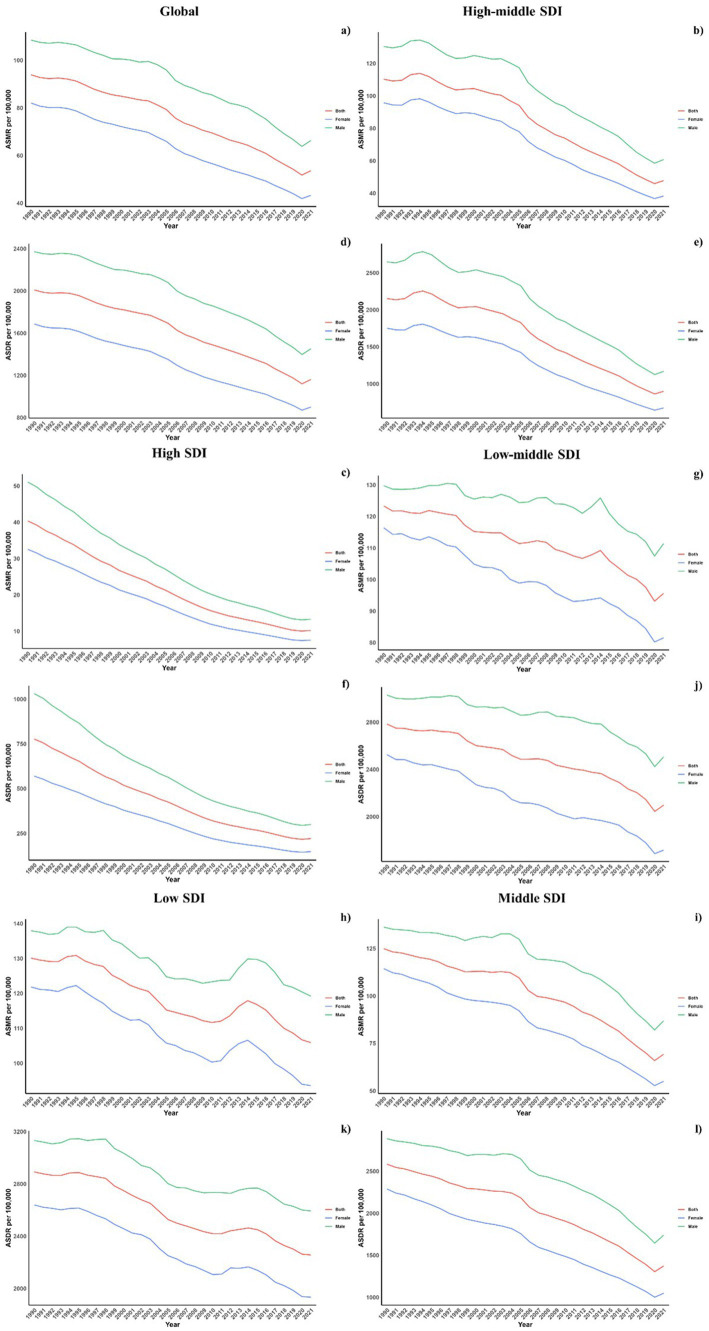
Sex disparity in CVDs burden attributable to air pollution across SDI regions.

Across all SDI regions, males reported higher mortality and DALY rates, with the gender disparity persisting uniformly across these regions (Figure 6).

### 3.3 Association with the socio-demographic index

**Figure 7:**
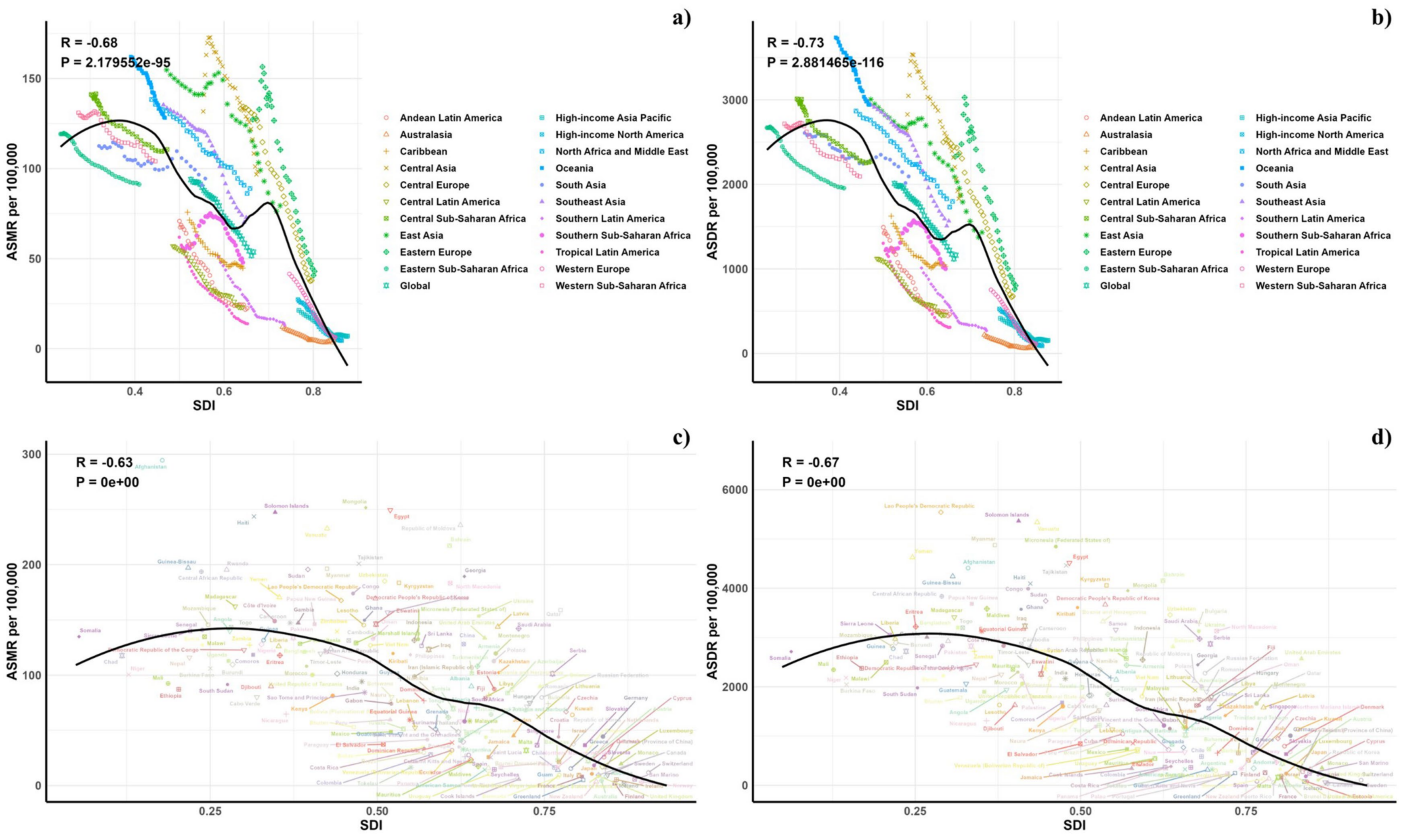
Correlations between ASMR (a,c) and ASDR (b,d) of CVDs attributable to air pollution and SDI at the regional level (a,b) and the national level (c,d).

Figure 7 illustrates the comparison between the observed and projected age-standardized DALY (ASDR) and mortality rates (ASMR) attributable to air pollution against socio-demographic index (SDI) values at both regional and national levels from 1990 to 2021. An inverse relationship was noted between ASDR and SDI, suggesting a decline in burden as SDI increased. Regions such as Eastern Europe, Oceania, Central Asia, Central Europe, North Africa, East Asia, the Middle East, and Southeast Asia displayed higher than expected ASDR during this period. Additionally, observed ASDR values exceeded projections in Central Latin America, Tropical Latin America, Southern Latin America, and the Caribbean. The pattern for observed versus expected ASMR based on SDI at regional levels mirrored the ASDR findings. Figure 7 also depict the observed versus expected ASDR and ASMR at the national level for 2019, showing a similar inverse correlation between these rates and SDI values, both regionally and nationally.

### 3.4 Decomposition analysis of the change in DALYs

**Figure 8:**
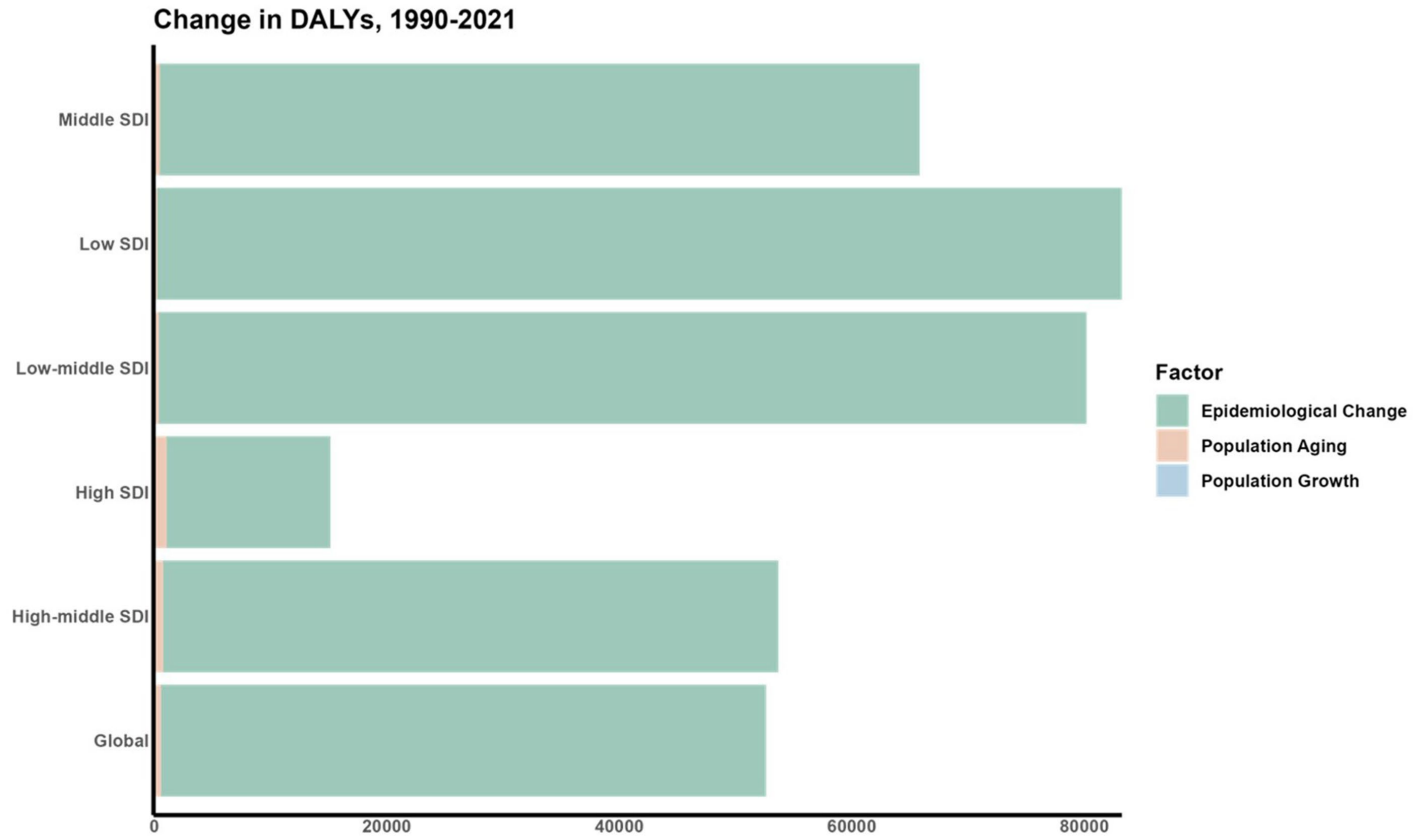
Decomposition analysis of the change in DALYS.

A decomposition analysis was conducted to assess the impact of three key factors: aging populations, demographic growth, and shifts in epidemiology, on the variation in DALYs from 1990 to 2021 (Figure 8). In tracking the trends of ASDR, shifts in epidemiological factors were identified as major contributors to the fluctuations in DALYs for CVDs linked to air pollution across various SDI regions.

### 3.5 Forecasts for the mortality, DALYs rate, ASMR and ASDR of CVD attributable to air pollution in global (2022–2050)

**Figure 9:**
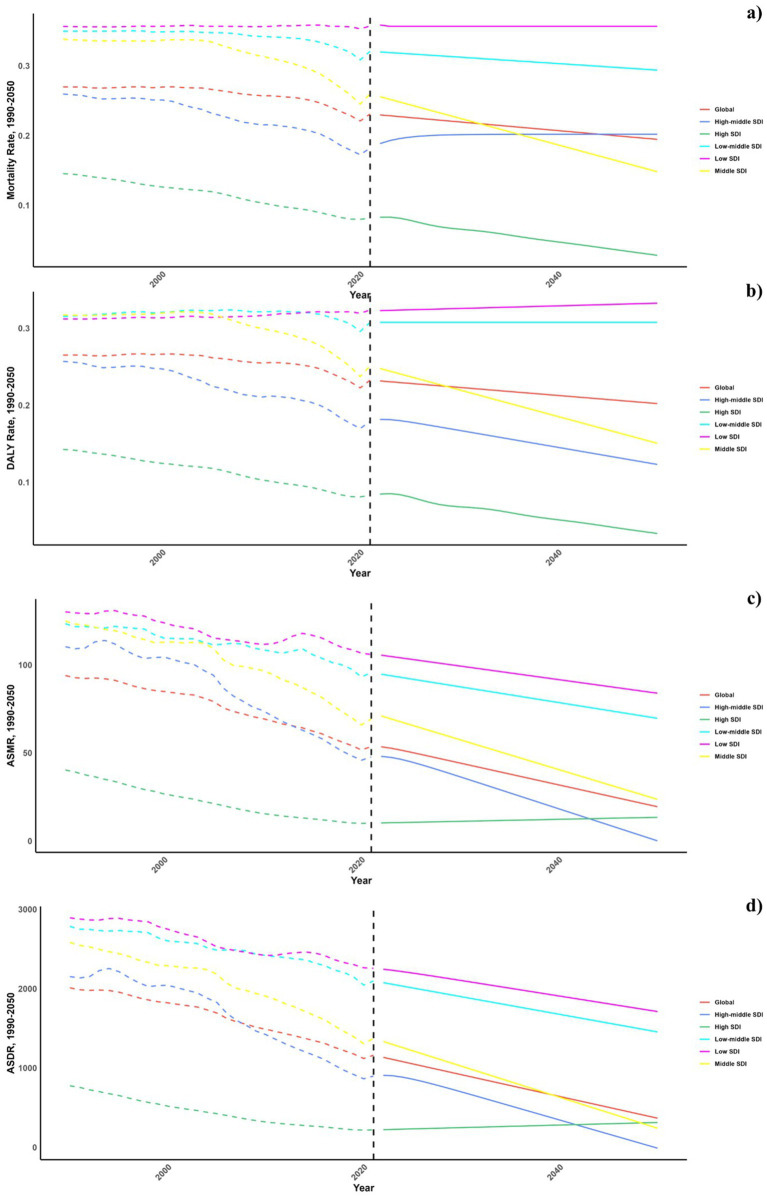
Estimated trends of mortality rate (a), DALYs rate (b), ASMR (c) and ASDR (d), 1990–2050 at the regional level.

**Figure 10:**
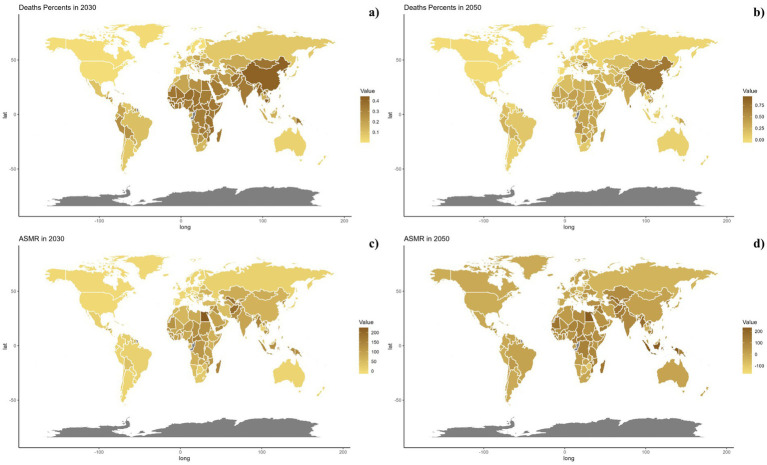
Estimated trends of mortality rate (a,b) and ASMR (c,d) in 2030 (a,c) and 2050 (b,d) at the national level.

**Figure 11:**
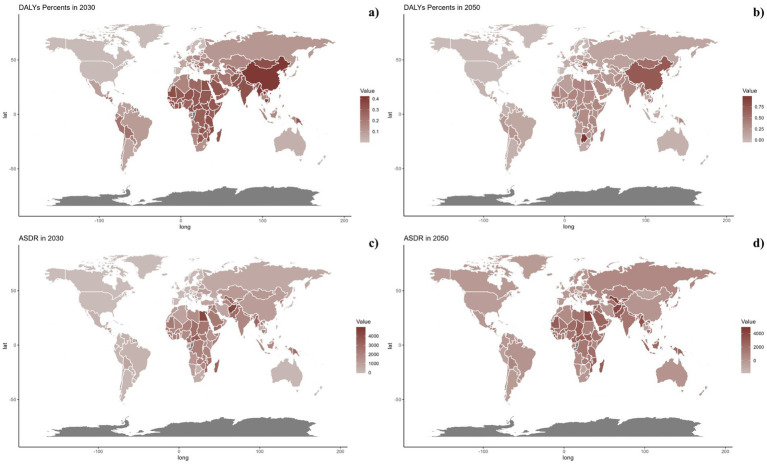
Estimated trends of DALYs rate (a,b) and ASDR (c,d) in 2030 (a,c) and 2050 (b,d) at the national level.

Forecasts for mortality rates, DALY rates, ASMR, and ASDR of CVDs linked to air pollution are presented in Figures 9–11. At the regional level, increases are anticipated in both low and high-middle SDI regions for mortality and DALYs rate, whereas only the high SDI regions are expected to see rises in ASDR and ASMR. Nationally, the distribution is projected to remain consistent in 2030 and 2050. Nonetheless, the projected burden of CVDs due to air pollution is significantly greater in China compared to other countries for both 2030 and 2050.

## 4 Discussion

This research evaluated the global burden of cardiovascular diseases (CVD) attributable to air pollution from 1990 to 2021, uncovering a significant decline over the period studied. The age groups most impacted were those below 75–79 and above 80, with males showing higher incidence rates. A decomposition analysis revealed that epidemiological shifts were the primary factors driving these changes. Predictions indicate increasing burdens in regions with low and high-middle socio-demographic index (SDI), along with rises in age-standardized death rate (ASDR) and age-standardized mortality rate (ASMR) in high SDI areas. To our knowledge, this is the first comprehensive assessment of the CVD impact from air pollution.

Key factors contributing to health-related adverse effects from air pollution include ozone and fine particulate matter. These pollutants affect bodily systems through mechanisms like oxidative stress, inflammation, and disturbances in endothelial and autonomic functions. The cardiovascular health impacts of air pollution can be traced from the initial physiological responses to pollutants, through their transmission, to their effects on target organs (23).

Primary reactions occurring in the lungs involve oxidative stress, local inflammation, and receptor activations initiated by pollutants such as PM_2.5_. These pollutants are ingested by alveolar macrophages through respiratory phagocytosis, leading to prolonged inflammatory responses in other tissues. The presence of pollutants activates inflammatory pathways involving NOD-like and Toll-like receptors, and the generation of reactive oxygen species (24–29).

Exposure to air pollution has been linked to systemic inflammation, primarily driven by the production of biological intermediates such as oxidized phospholipids and 7-ketocholesterol. These intermediates activate the TLR4, NOX2, and NCF1 pathways, leading to vascular and systemic inflammation (28). Furthermore, 7-ketocholesterol plays a role in promoting thrombosis, atherosclerosis, and endothelial dysfunction through CD36 pathways (30). Prolonged inhalation of PM_2.5_ escalates the production of reactive oxygen species (ROS) and inflammatory infiltration in the vasculature, potentially resulting in diastolic dysfunction, increased cardiac afterload, and compromised coronary reserve, which can culminate in cardiac complications such as left ventricular hypertrophy and fibrosis.

*In vivo* studies show that PM_2.5_ affects plasma tissue plasminogen activator levels, influencing thrombus formation. Variability in the composition and concentration of air pollution may explain discrepancies in research findings. PM_2.5_ also impacts the central nervous system, potentially causing autonomic dysfunction or adrenal activation through stimulation of the olfactory nerve (31–33). Exposure to ozone may similarly activate the adrenal axis (34), potentially leading to conditions like insulin resistance or hypertension. Moreover, while PM_2.5_ is known to induce thrombo-inflammatory responses and affect DNA methylation, its complete impact on genomic methylation and chromatin structure is yet to be fully elucidated (35, 36).

Chronic exposure to environmental pollutants can lead to lasting organ changes, thereby increasing vulnerability to cardiometabolic diseases such as diabetes, hypertension, and renal disease, while also accelerating atherosclerosis (37). Studies have linked even low-level, prolonged exposure to PM_2.5_ with a heightened risk of cardiovascular mortality, noting that increases in PM_2.5_ levels are associated with rising risks of cardiovascular death (38–41). In contrast, ozone exposure exhibits a weaker connection with CVD mortality (42). Additionally, prolonged exposure to PM_2.5_ correlates with greater carotid intima-media thickness, which suggests advancing atherosclerosis and elevated blood pressure, though these relationships remain subject to debate (23, 43–47). PM_2.5_ exposure also heightens the risk of severe cardiac events in individuals with pre-existing heart conditions and contributes to the development of diabetes and insulin resistance (29, 48–50).

Previous studies have highlighted significant variations in the CVD burden from air pollution across BRICS nations (51). In 2019, Brazil’s age-standardized rate of CVD attributable to air pollution was approximately five times lower than India’s. During the observation period, Brazil saw the most significant decrease in CVD burden from air pollution and household air pollution (HAP) from solid fuels. Brazilian authorities have implemented measures to reduce emissions from both stationary and mobile sources, leading to a noticeable decline in air pollutants from 1996 to 2009 (52, 53). Initiatives like the Family Health Program and integrated approaches to managing non-communicable diseases have also contributed to reducing the CVD burden (53, 54).

Russia has seen a notable reduction in the CVD burden, especially from ambient particulate matter pollution (51). Since 1990, the WHO European Centre for Environment and Health launched programs to improve air quality and control pollution, including monitoring its health effects in the European Region, which includes Russia (55). In 2005, WHO organized a workshop in Eastern Europe, the Caucasus, and Central Asia (EECCA) to develop strategies to mitigate air pollution’s health impacts in EECCA nations, including Russia (56).

Conversely, South Africa has experienced a rising trend in all-ages CVD fatalities and a slow decline in age-standardized CVD mortality linked to air pollution (51). A recent report highlighted that pollution continues to pose significant health and economic risks in low-and middle-income countries (LMICs), with ambient air pollution remaining the main cause of pollution-related illnesses and deaths in Africa. Consequently, the incidence of non-communicable disease (NCD) deaths related to air pollution is rising in many African nations (57–59).

To combat CVDs, controlling air pollution is critical. Governments should enforce air quality standards and establish mechanisms for monitoring, enforcing, and ensuring accountability in pollution management. Technological advances in industry and sustainable energy initiatives like solar power are essential. Researchers should focus on creating and evaluating methods to mitigate air pollution’s effects on cardiovascular health. Public education on pollution risks and personal health measures such as using air filters and consuming antioxidants are necessary. Future strategies might include integrating data from multiple environmental sources and fostering cross-disciplinary research to refine pollution risk assessments. Clinicians should guide those at risk to maintain a diet rich in antioxidants and fresh produce.

The COVID-19 pandemic’s lockdown measures significantly impacted air quality in various regions around the world. Research shows that due to reduced traffic flow and the suspension of certain industrial activities, many cities experienced a marked improvement in air pollution levels in a short period. For instance, in 2020, several major cities globally reported significant reductions in the concentrations of nitrogen oxides (NO*x*) and fine particulate matter (PM_2.5_). This improvement in air quality, particularly among the working-age population that is at high risk for cardiovascular diseases, may have helped reduce the incidence of cardiovascular events (60).

Further studies indicate a close association between improved air quality and the reduction in acute cardiovascular events. For example, during the pandemic, there was a decrease in emergency room visits and hospitalizations for various cardiovascular diseases, although this may also be influenced by changes in people’s healthcare-seeking behaviors during the pandemic. Additionally, the reduction in outdoor activities during the pandemic could have lessened the health risks associated with air pollution for individuals with respiratory and cardiovascular conditions (61).

However, the positive impacts brought about by the lockdown measures may be temporary. As economic activities gradually resume, air pollution levels could quickly return to pre-pandemic levels or even exceed them in some areas. Monitoring and researching air quality in the post-pandemic and post-pandemic periods will be crucial for understanding long-term trends and developing appropriate responses (62).

Our study faces a few significant limitations. First, there is a lack of primary data from underdeveloped regions, especially in Sub-Saharan Africa, where estimates were largely derived through mathematical models, resulting in broad uncertainty ranges. Second, air pollution consists of a complex blend of multiple components, each with distinct physicochemical characteristics and toxicological effects, which differ widely by geographic area and season.

## 5 Conclusion

This research conducted a detailed evaluation of the global CVD burden due to air pollution from 1990 to 2021, noting a substantial reduction in this burden over time. The most affected age groups were those under 75–79 and those over 80, with men experiencing a higher impact. The analysis indicated that changes in epidemiology were the primary drivers of these trends. Moreover, future projections suggest an increase in both ASDR and ASMR in low and high-middle SDI regions, with only high SDI regions expected to see rises.

This highlights a critical need for policymakers to develop and enhance targeted preventive strategies for specific demographic groups to mitigate the CVD burden linked to air pollution moving forward.

## Data Availability

The raw data supporting the conclusions of this article will be made available by the authors, without undue reservation.
