# Supplementary material for: Effect of air pollution on the global burden of cardiovascular diseases and forecasting future trends of the related metrics: a systematic analysis from the Global Burden of Disease Study 2021
Source: Front Med (Lausanne). 2024 Oct 11;11:1472996. doi: 10.3389/fmed.2024.1472996 (PMC11502364; doi:10.3389/fmed.2024.1472996)
Supplement: Supplementary file 1 [file Table_1.docx]

Table S1 Global and regional deaths and DALYs of CVDs attributable to air pollution in 1990 and 2021, and EAPC of ASDR and ASMR from 1990 to 2021 in 204 nations.

| Location | Deaths Number in 1990 | Deaths Number in 2021 | ASMR in 2021 | EPAC, 1999-2021 | DALY Number in 1990 | DALY Number in 2021 | ASDR in 2021 | EPAC, 1999-2021 |
| --- | --- | --- | --- | --- | --- | --- | --- | --- |
| China | 969368.982 (1153392.5738, 796427.1291) | 1467819.3363 (1870488.58, 1115199.9553) | 80.2729 (101.6316, 61.1427) | -2.0482 (-1.6767, -2.4183) | 23431250.875 (27895766.9911, 19172805.2634) | 28445610.4492 (36385479.1104, 21700368.0893) | 1432.8498 (1826.6884, 1100.8764) | -2.3384 (-2.0107, -2.6650) |
| Democratic People's Republic of Korea | 20470.2325 (25987.7908, 15622.4344) | 43989.0876 (55184.9921, 34051.8565) | 146.4569 (184.1631, 112.2008) | -0.2510 (-0.0604, -0.4413) | 518859.4402 (674357.954, 395074.7627) | 1028189.1811 (1301533.1949, 786485.9835) | 3171.1241 (4011.9804, 2438.2198) | -0.1628 (0.0188, -0.3441) |
| Taiwan (Province of China) | 5795.8903 (10088.4234, 2496.3186) | 4300.8223 (5416.1067, 3297.4894) | 9.8220 (12.3850, 7.5678) | -4.5179 (-4.2219, -4.8131) | 138219.4765 (242582.8889, 58751.0525) | 92424.7944 (114983.8906, 72701.1805) | 222.9873 (277.2028, 175.6988) | -4.0837 (-3.7996, -4.3670) |
| Cambodia | 6400.6543 (7637.288, 5227.4092) | 12653.492 (15523.6323, 9556.7281) | 130.3558 (157.9727, 100.0271) | -1.0370 (-0.9708, -1.1032) | 170563.8802 (204145.643, 139149.9599) | 304995.0908 (383706.4749, 227702.4839) | 2591.6896 (3201.3333, 1975.3013) | -1.4044 (-1.3327, -1.4761) |
| Indonesia | 108634.5209 (132721.7621, 85007.4459) | 156398.5 (212783.0044, 107307.8846) | 83.0524 (112.1290, 57.5077) | -1.4568 (-1.1030, -1.8094) | 3069824.0599 (3749492.549, 2451862.0937) | 4069821.205 (5559394.0354, 2800401.0802) | 1731.3833 (2344.7297, 1193.3650) | -1.7693 (-1.4314, -2.1061) |
| Lao People's Democratic Republic | 4552.4131 (5630.7097, 3591.6203) | 5755.9481 (7499.2698, 3972.5904) | 151.8194 (194.4992, 106.4069) | -1.8582 (-1.7981, -1.9181) | 126310.9777 (158803.1759, 98667.0491) | 148618.4506 (197307.6908, 100637.8232) | 3195.0592 (4157.2303, 2191.0091) | -2.1729 (-2.1056, -2.2401) |
| Malaysia | 6786.6353 (10481.1097, 3298.3872) | 9683.4233 (13366.2942, 6540.3015) | 38.6500 (53.2690, 26.1973) | -2.5821 (-2.3544, -2.8092) | 168514.4181 (260581.8467, 82339.714) | 234048.4631 (321404.488, 156970.9656) | 830.5487 (1144.1633, 559.6716) | -2.6783 (-2.4451, -2.9110) |
| Maldives | 118.2213 (141.6044, 90.9234) | 49.7077 (77.4756, 27.784) | 17.4665 (27.2985, 9.7479) | -8.3563 (-7.9491, -8.7616) | 3475.1718 (4302.9183, 2678.0098) | 1171.7324 (1826.0573, 650.9959) | 331.3023 (519.0493, 185.3105) | -8.9974 (-8.5576, -9.4351) |
| Myanmar | 46339.0969 (57767.651, 36656.8984) | 57131.7745 (73548.1404, 42445.5973) | 136.7202 (176.0094, 102.3439) | -1.9700 (-1.8072, -2.1324) | 1264221.8817 (1585503.474, 992106.6789) | 1367108.5288 (1766396.7447, 1013690.2922) | 2860.3659 (3678.8292, 2126.4424) | -2.2809 (-2.0983, -2.4631) |
| Philippines | 29502.5366 (34860.0168, 24434.4884) | 60328.6203 (76147.869, 44103.5911) | 84.4575 (106.0281, 61.5122) | -1.3036 (-1.1322, -1.4747) | 792675.6514 (938702.8517, 657367.1775) | 1594472.3844 (2025320.9846, 1164965.2831) | 1892.2250 (2396.4128, 1382.3514) | -1.0813 (-0.8996, -1.2627) |
| Sri Lanka | 11982.0443 (13771.3626, 10268.4332) | 12764.8738 (21258.3015, 6572.8334) | 53.9805 (89.2746, 27.7445) | -2.7503 (-2.2779, -3.2204) | 279323.8291 (323265.5304, 237578.4116) | 265148.7237 (442347.415, 137718.1644) | 1029.6348 (1721.7724, 539.1578) | -2.9692 (-2.4977, -3.4384) |
| Thailand | 25399.9493 (31042.2525, 19436.2043) | 30929.1698 (41775.3942, 22096.4079) | 28.7756 (38.8778, 20.6126) | -4.3177 (-3.9519, -4.6821) | 645525.3988 (795366.968, 491515.7085) | 697750.6134 (936055.1142, 501043.3711) | 667.7611 (895.8098, 481.4765) | -3.9979 (-3.6425, -4.3519) |
| Timor-Leste | 324.0177 (400.2074, 251.0896) | 937.1694 (1260.5193, 584.1596) | 129.3173 (174.0153, 80.7545) | -0.6054 (-0.4446, -0.7659) | 9224.5924 (11497.3219, 7060.6739) | 22034.4879 (29678.9683, 13736.9849) | 2647.6512 (3542.3054, 1654.2052) | -0.7892 (-0.6062, -0.9718) |
| Viet Nam | 51696.1815 (64894.7342, 39535.7671) | 67067.0236 (86043.231, 47297.8228) | 79.5006 (101.3385, 56.5900) | -1.9294 (-1.7046, -2.1536) | 1155795.2295 (1456944.6144, 879853.2976) | 1478796.5462 (1919784.3315, 1044904.9318) | 1554.7047 (2004.6969, 1105.3314) | -2.0436 (-1.8384, -2.2484) |
| Fiji | 466.619 (607.314, 302.213) | 458.7637 (813.0848, 148.1071) | 73.7132 (128.7420, 24.1705) | -2.4734 (-2.2584, -2.6879) | 14135.8583 (18398.7341, 9070.2148) | 12412.1911 (22133.7637, 3931.1915) | 1619.6205 (2872.3999, 518.0555) | -2.6159 (-2.4429, -2.7887) |
| Kiribati | 59.1876 (71.2676, 47.1653) | 75.913 (100.2583, 56.2177) | 122.3802 (158.4607, 91.9498) | -1.3331 (-1.1306, -1.5351) | 1829.0272 (2212.1678, 1465.6307) | 2376.1601 (3185.8218, 1740.0565) | 2993.9114 (3950.3091, 2218.7149) | -1.4330 (-1.2329, -1.6328) |
| Marshall Islands | 19.7715 (27.2243, 13.719) | 32.0211 (48.2556, 21.2757) | 111.6297 (162.9092, 75.4039) | -0.4378 (-0.2557, -0.6195) | 568.1259 (780.3529, 391.7071) | 1011.2348 (1526.8733, 673.7494) | 2634.5580 (3917.0275, 1771.2446) | -0.3931 (-0.2058, -0.5800) |
| Micronesia (Federated States of) | 88.2401 (111.9901, 66.0555) | 70.1907 (103.9878, 44.3139) | 113.0268 (165.3850, 71.7174) | -1.9502 (-1.9113, -1.9891) | 2455.3172 (3133.3249, 1822.4891) | 2076.7195 (3087.3805, 1307.8172) | 2668.1585 (3946.6863, 1703.6695) | -1.9658 (-1.9277, -2.0039) |
| Papua New Guinea | 2435.2346 (3232.3658, 1794.6882) | 5901.8994 (7766.281, 4226.3905) | 143.2119 (185.8379, 103.8893) | -0.4607 (-0.3934, -0.5279) | 72095.2836 (97182.3798, 52312.6856) | 171986.5453 (228421.9704, 121951.6173) | 3201.1770 (4192.3412, 2311.4941) | -0.5510 (-0.4910, -0.6109) |
| Samoa | 121.0945 (146.6908, 95.0067) | 162.2074 (217.0942, 108.0188) | 127.9249 (170.5715, 86.1713) | -0.8088 (-0.7523, -0.8652) | 3086.4461 (3783.4375, 2384.7673) | 4119.4854 (5549.4375, 2727.3605) | 2838.5652 (3807.8747, 1892.1535) | -0.7473 (-0.6850, -0.8095) |
| Solomon Islands | 281.7173 (364.1739, 198.7551) | 656.6661 (851.3763, 501.2473) | 230.6303 (291.6115, 179.0788) | -0.3961 (-0.2986, -0.4935) | 8179.1699 (10796.6367, 5410.7172) | 18931.2281 (24796.7066, 14379.6348) | 5194.0651 (6679.0335, 4018.2458) | -0.3847 (-0.2673, -0.5019) |
| Tonga | 44.0586 (54.6922, 34.1054) | 43.7077 (63.2728, 26.4568) | 56.9549 (82.1380, 34.5176) | -1.3363 (-1.1914, -1.4810) | 1132.2718 (1402.4575, 882.2418) | 1000.6987 (1475.949, 608.7987) | 1244.1775 (1826.0178, 757.5829) | -1.4523 (-1.3209, -1.5835) |
| Vanuatu | 130.002 (164.3258, 100.8531) | 316.6882 (381.8796, 249.8253) | 216.3345 (258.1585, 172.8357) | -0.5755 (-0.5305, -0.6204) | 3923.2978 (5033.9677, 2972.7758) | 9419.7089 (11452.929, 7354.1048) | 5054.5733 (6101.4687, 4006.8488) | -0.5893 (-0.5290, -0.6496) |
| Armenia | 2764.1697 (4046.8595, 1636.4781) | 3338.9715 (4427.5458, 2294.6625) | 77.5780 (102.9148, 53.3281) | -2.0847 (-1.8259, -2.3429) | 57883.3393 (84286.4511, 33984.7298) | 61127.441 (81137.9617, 42396.0552) | 1427.1951 (1895.3706, 990.5066) | -2.0817 (-1.8608, -2.3021) |
| Azerbaijan | 5669.1311 (8485.2387, 2870.0679) | 6564.6366 (10127.7469, 3549.1516) | 83.8336 (128.9953, 45.3611) | -1.7668 (-1.4838, -2.0490) | 129130.7749 (194430.1731, 64538.8456) | 140384.5742 (218148.2254, 75527.2914) | 1529.8659 (2368.2234, 828.5039) | -2.3237 (-2.0042, -2.6422) |
| Georgia | 7381.5677 (11358.7248, 3443.2109) | 3498.4237 (4969.1748, 2169.3394) | 54.4661 (77.3068, 34.2346) | -3.7938 (-3.0409, -4.5408) | 151814.1438 (234484.2312, 69839.1872) | 64267.3992 (92217.3678, 40436.0017) | 1076.6945 (1559.1817, 676.2879) | -3.9583 (-3.1028, -4.8062) |
| Kazakhstan | 11060.5667 (18070.5514, 4852.2217) | 10153.8576 (13673.4319, 7087.7525) | 74.0623 (99.6375, 51.6289) | -2.0960 (-1.5772, -2.6120) | 243991.3145 (401482.7816, 107081.7922) | 208207.0304 (280560.4637, 143985.2656) | 1297.5877 (1743.5417, 902.0771) | -2.6075 (-2.0164, -3.1950) |
| Kyrgyzstan | 4077.7205 (5172.8169, 3040.3494) | 4297.7035 (5431.4463, 3226.4968) | 112.9039 (142.7662, 84.7654) | -1.0722 (-0.6106, -1.5316) | 90182.7413 (114619.3774, 67015.4509) | 95164.5366 (120287.7851, 72317.4674) | 2125.1288 (2678.6234, 1608.4701) | -1.5114 (-0.9857, -2.0343) |
| Mongolia | 1988.2302 (2354.7798, 1624.4568) | 1870.1559 (2523.4542, 1303.5605) | 109.0725 (146.6512, 75.6026) | -2.8315 (-2.3643, -3.2965) | 45658.0155 (54286.3658, 37348.2339) | 46004.3324 (61814.5835, 31903.9911) | 2104.1813 (2827.8766, 1466.6074) | -2.9453 (-2.4450, -3.4429) |
| Tajikistan | 4699.3343 (5680.9227, 3694.4435) | 5684.6113 (7095.1225, 4375.7104) | 136.6317 (170.9367, 105.0439) | -1.4961 (-1.1312, -1.8596) | 103833.1606 (125053.1957, 82708.736) | 131436.8507 (164076.5705, 101660.3338) | 2538.2233 (3152.0105, 1964.9155) | -1.8037 (-1.4703, -2.1359) |
| Turkmenistan | 1466.2802 (2720.5873, 435.3611) | 3064.4621 (4724.2779, 1795.5532) | 91.5037 (141.0180, 53.7148) | -0.6397 (-0.3353, -0.9432) | 34090.5621 (63398.6514, 10078.4296) | 70856.8428 (109637.837, 41233.0146) | 1842.2543 (2840.9290, 1076.6980) | -0.5928 (-0.2668, -0.9177) |
| Uzbekistan | 15445.0618 (20737.4923, 10502.6464) | 27126.3122 (35139.7504, 19586.7242) | 134.9665 (175.1373, 97.4554) | -0.2898 (0.0677, -0.6461) | 339315.9519 (449264.1634, 234538.3887) | 613697.6125 (795653.994, 448857.7503) | 2556.0783 (3302.7895, 1869.3574) | -0.6014 (-0.2517, -0.9500) |
| Albania | 2532.4082 (2994.3385, 2045.7929) | 2230.6988 (3336.0008, 1410.0814) | 54.7468 (81.7546, 34.6149) | -3.3462 (-3.0194, -3.6719) | 50567.6028 (59584.6033, 40648.7252) | 36133.6792 (54402.6433, 22906.8627) | 857.1307 (1288.2704, 543.7697) | -3.7565 (-3.4311, -4.0808) |
| Bosnia and Herzegovina | 5366.2823 (6170.6838, 4534.7891) | 4422.9076 (6004.7026, 3115.4005) | 69.2235 (93.9618, 48.7326) | -3.1413 (-2.9524, -3.3299) | 120224.7983 (137730.9012, 102129.6851) | 77298.5589 (105189.3447, 54745.3715) | 1234.3304 (1678.2662, 874.3808) | -3.4213 (-3.2638, -3.5785) |
| Bulgaria | 16233.8637 (22988.3421, 9942.4968) | 9160.9455 (13317.668, 6832.6725) | 64.2677 (93.4068, 48.0675) | -3.8669 (-3.4622, -4.2699) | 333735.5707 (473493.489, 203424.1782) | 161350.8551 (234372.7074, 119876.4668) | 1175.0884 (1706.3404, 872.3163) | -3.7378 (-3.3369, -4.1370) |
| Croatia | 5668.0261 (8392.5747, 3085.9922) | 2816.1778 (3602.0557, 2070.0674) | 28.4316 (36.4072, 20.9039) | -4.3738 (-4.1336, -4.6134) | 106522.7717 (156179.3899, 57873.5695) | 43576.0407 (55751.0887, 32047.4057) | 468.6940 (600.7801, 344.4137) | -4.5212 (-4.2602, -4.7815) |
| Czechia | 14602.2598 (21874.5589, 7935.2595) | 5056.9893 (6503.7183, 3641.5604) | 21.6231 (27.8328, 15.5998) | -4.8982 (-4.5526, -5.2426) | 281155.8015 (418959.8369, 153680.6459) | 81948.1187 (104938.4635, 59876.6843) | 370.8346 (475.8474, 272.5000) | -5.2289 (-4.9289, -5.5280) |
| Hungary | 15076.5715 (22682.4187, 8271.8857) | 6263.1383 (9373.8228, 4416.111) | 29.2411 (43.7210, 20.8049) | -4.2968 (-4.0349, -4.5581) | 308883.9771 (464624.0682, 170089.4979) | 106555.9626 (158204.454, 76097.482) | 537.8748 (795.6739, 383.8663) | -4.6271 (-4.3888, -4.8649) |
| North Macedonia | 2981.6029 (3726.2741, 2086.5204) | 2665.4466 (3460.4751, 1998.1267) | 112.1339 (143.6987, 84.8333) | -2.0111 (-1.3612, -2.6567) | 62806.5719 (78200.202, 44198.533) | 49575.1305 (64619.7074, 37204.0863) | 1753.6767 (2265.4658, 1322.5447) | -2.6497 (-2.1188, -3.1778) |
| Montenegro | 604.2163 (871.155, 345.0244) | 694.7841 (1179.6414, 473.0096) | 82.2236 (139.5705, 56.0030) | -1.0381 (-0.7613, -1.3142) | 12240.7496 (17567.431, 7048.1276) | 12029.1289 (20228.4765, 8243.1334) | 1319.6058 (2225.0664, 906.2879) | -1.6950 (-1.3699, -2.0190) |
| Poland | 55153.1981 (72352.5541, 36737.5578) | 23353.6297 (30123.5044, 17798.0925) | 29.9880 (38.7338, 22.8228) | -5.0924 (-4.9022, -5.2823) | 1124428.9984 (1479042.7673, 741554.7054) | 392816.3819 (502916.3656, 298754.4323) | 537.8214 (687.9272, 408.6593) | -5.3269 (-5.1450, -5.5085) |
| Romania | 31436.291 (44455.1033, 18448.5219) | 15547.2949 (22171.2596, 11167.3913) | 38.0819 (54.3548, 27.3514) | -4.9057 (-4.5223, -5.2876) | 627851.4323 (886575.0105, 368846.8497) | 263436.8113 (375988.674, 188792.4804) | 696.6962 (995.0285, 497.7036) | -4.9257 (-4.5073, -5.3423) |
| Serbia | 17286.3044 (22869.644, 10640.0567) | 12658.3211 (17270.5018, 9330.3548) | 73.0737 (99.8054, 53.8318) | -4.3300 (-3.9007, -4.7574) | 336684.8357 (448623.2204, 207736.5002) | 206902.4005 (281893.3707, 153997.7092) | 1228.8787 (1675.3007, 912.2915) | -4.2409 (-3.7999, -4.6799) |
| Slovakia | 6226.4611 (9243.6797, 3357.1369) | 3469.1334 (4457.2195, 2569.8586) | 36.5247 (46.9983, 27.0436) | -3.3423 (-3.0881, -3.5957) | 125474.1033 (186695.2006, 68834.0271) | 60126.5496 (76616.3298, 44661.4883) | 634.5610 (809.1734, 470.8683) | -3.7841 (-3.5199, -4.0475) |
| Slovenia | 1288.5774 (1942.9263, 699.2492) | 516.7381 (684.9432, 363.7216) | 9.5654 (12.7176, 6.7810) | -5.7097 (-5.5055, -5.9135) | 24738.6786 (37504.899, 13437.2472) | 8126.5399 (10837.1497, 5746.6937) | 169.7816 (225.5418, 120.3206) | -5.9631 (-5.7613, -6.1645) |
| Belarus | 15648.6738 (22505.0221, 8777.7655) | 10402.4491 (14405.7424, 7205.4648) | 63.5393 (88.0308, 43.9902) | -2.9375 (-2.5044, -3.3686) | 306268.714 (440131.5582, 171246.7422) | 189840.6019 (262715.5145, 130432.0512) | 1187.2083 (1642.7610, 815.2396) | -3.0894 (-2.5762, -3.6000) |
| Estonia | 1691.7235 (2787.3835, 704.1881) | 173.573 (342.9987, 53.5192) | 5.3295 (10.5323, 1.6483) | -10.1794 (-9.5253, -10.8288) | 31370.586 (51647.8542, 12847.485) | 2686.3812 (5306.5468, 833.449) | 93.8126 (185.3615, 29.3270) | -10.3226 (-9.6755, -10.9650) |
| Latvia | 4984.8039 (7141.1348, 2822.0668) | 1354.7577 (2016.4023, 889.0709) | 29.1494 (43.5034, 19.0818) | -6.0520 (-5.5854, -6.5162) | 94239.8828 (135281.9759, 53192.1736) | 21812.5116 (32753.4396, 14394.7632) | 526.4608 (794.7816, 348.0964) | -6.3002 (-5.7861, -6.8115) |
| Lithuania | 4452.09 (6813.8475, 2301.9094) | 1504.7645 (2158.0749, 904.4438) | 22.1411 (31.8059, 13.3251) | -5.3547 (-5.0127, -5.6954) | 82619.2924 (126608.5336, 42991.524) | 23317.1816 (33367.7411, 14045.1199) | 384.9489 (550.5026, 231.6669) | -5.5224 (-5.1445, -5.8987) |
| Republic of Moldova | 7647.7876 (9279.7896, 6015.6165) | 3297.4766 (4713.9577, 2112.2676) | 54.5418 (77.9266, 34.8976) | -5.6088 (-5.2054, -6.0105) | 156988.8746 (189884.534, 123752.6889) | 63774.2407 (90694.464, 40838.585) | 1071.6904 (1523.2770, 686.2698) | -5.2465 (-4.7999, -5.6910) |
| Russian Federation | 182820.6247 (276721.2658, 88230.404) | 78923.9169 (123535.2208, 47591.5202) | 32.7946 (51.3169, 19.7922) | -4.9459 (-4.3578, -5.5304) | 3719363.2175 (5635396.6333, 1784797.427) | 1481512.2004 (2323673.7029, 883070.8353) | 627.7251 (983.9381, 374.1215) | -4.9535 (-4.3135, -5.5892) |
| Ukraine | 93297.9578 (136214.7216, 50490.8961) | 57816.4163 (91316.4259, 33014.8119) | 72.6376 (114.9186, 41.4813) | -3.1674 (-2.5893, -3.7421) | 1762644.3894 (2574604.3181, 950402.0659) | 1014563.6865 (1568077.4896, 584272.3159) | 1303.4824 (2010.9901, 749.5298) | -3.1100 (-2.5332, -3.6835) |
| Brunei Darussalam | 13.7329 (34.8415, 0.8883) | 19.7854 (39.3001, 4.2095) | 7.6838 (15.1519, 1.6084) | -1.8367 (-1.0378, -2.6293) | 367.7647 (922.9541, 23.7565) | 556.3971 (1105.5644, 118.9952) | 158.0348 (311.4226, 33.2569) | -2.1215 (-1.3257, -2.9109) |
| Japan | 24333.3371 (49909.9218, 5004.2492) | 27456.1568 (41949.8227, 14559.1623) | 5.5050 (8.2921, 2.9625) | -3.5658 (-3.2076, -3.9227) | 467318.4361 (960621.0853, 94752.1759) | 439292.8649 (661705.1765, 231891.9559) | 120.9197 (181.5853, 65.8124) | -2.9617 (-2.6392, -3.2832) |
| Republic of Korea | 12650.75 (21780.7479, 5237.9871) | 13526.2622 (18256.0725, 9137.8193) | 14.9626 (20.2345, 10.1144) | -4.9587 (-4.6621, -5.2544) | 309693.7068 (530701.1554, 128143.016) | 253065.6896 (337263.2372, 174460.6813) | 278.8934 (371.6296, 192.2248) | -4.9972 (-4.6998, -5.2937) |
| Singapore | 1252.248 (2031.3118, 499.536) | 798.4332 (1186.452, 437.1036) | 9.5771 (14.2406, 5.2398) | -5.9359 (-5.5643, -6.3060) | 31199.047 (50689.7083, 12363.2672) | 17620.7976 (26129.8837, 9393.8579) | 206.6314 (306.4227, 110.2368) | -5.9397 (-5.5271, -6.3505) |
| Australia | 2307.2702 (6352.9487, 74.4783) | 2416.077 (3550.9616, 1403.885) | 4.5832 (6.7364, 2.6631) | -3.8780 (-3.3218, -4.4309) | 42647.1526 (116614.7705, 1349.7655) | 38421.3868 (55951.415, 22571.9422) | 82.1180 (119.2735, 48.2908) | -3.8671 (-3.2663, -4.4642) |
| New Zealand | 421.7729 (1247.0963, 9.7773) | 375.183 (631.8004, 149.1032) | 4.0579 (6.8274, 1.6161) | -3.7634 (-3.4308, -4.0950) | 8164.2797 (24004.856, 199.8395) | 6054.6326 (10147.1973, 2435.594) | 70.6259 (118.0624, 28.3482) | -4.1073 (-3.7159, -4.4971) |
| Andorra | 8.9505 (15.9306, 3.5345) | 7.0553 (11.301, 3.699) | 4.0096 (6.4437, 2.1114) | -4.6393 (-4.2822, -4.9951) | 188.0018 (330.0841, 73.625) | 116.7899 (187.5319, 61.7273) | 72.3077 (116.0853, 38.1472) | -4.8044 (-4.4565, -5.1510) |
| Austria | 6362.1197 (9848.591, 3344.3879) | 1966.6116 (2662.9348, 1360.8204) | 8.7376 (11.7990, 6.0883) | -6.0605 (-5.8199, -6.3006) | 110855.8097 (170436.4297, 58200.509) | 30316.3633 (41217.136, 21222.0907) | 153.1143 (206.8289, 107.5188) | -6.1658 (-5.9458, -6.3853) |
| Belgium | 6812.3946 (10701.0117, 3506.4983) | 1650.3833 (2196.0219, 1118.884) | 5.7336 (7.5881, 3.9045) | -6.4515 (-6.1260, -6.7760) | 119944.0221 (186690.5144, 61707.7033) | 26367.1789 (34942.8219, 18101.6251) | 107.0845 (140.7211, 73.8497) | -6.4126 (-6.1088, -6.7155) |
| Cyprus | 394.0649 (648.11, 188.252) | 300.201 (398.8169, 216.7028) | 17.8998 (23.7262, 12.8262) | -5.2179 (-5.0037, -5.4316) | 7102.8613 (11783.4815, 3384.4199) | 5140.3615 (6864.9291, 3668.5198) | 277.3499 (368.6643, 198.3208) | -5.0442 (-4.8203, -5.2675) |
| Denmark | 4141.8192 (6921.7672, 1934.084) | 670.6424 (959.7697, 404.2382) | 4.9449 (7.0852, 2.9829) | -7.7869 (-7.5063, -8.0667) | 71514.3477 (118264.1848, 33246.9699) | 10833.3629 (15469.5084, 6613.1083) | 88.6640 (126.5091, 54.1366) | -7.7789 (-7.5333, -8.0238) |
| Finland | 1469.7096 (3040.6312, 256.6207) | 338.5207 (737.7699, 43.1721) | 2.1475 (4.6508, 0.2754) | -7.5647 (-7.0661, -8.0607) | 28278.8781 (58074.4598, 4891.5811) | 5125.829 (11012.9846, 676.7943) | 37.7767 (80.5367, 4.9843) | -7.8902 (-7.4192, -8.3589) |
| France | 20814.768 (34975.2168, 9757.7553) | 7205.6499 (9997.0029, 4656.6272) | 3.7801 (5.2212, 2.4739) | -5.9469 (-5.6422, -6.2506) | 348261.7034 (577483.9987, 163508.3063) | 110235.611 (153615.1512, 73267.4924) | 71.8912 (99.8603, 48.1929) | -5.7062 (-5.4128, -5.9987) |
| Germany | 74447.8876 (117900.7805, 37992.6929) | 18382.3979 (25207.3024, 12239.4544) | 7.7398 (10.6003, 5.2001) | -6.3561 (-6.1404, -6.5713) | 1294904.0544 (2042885.5708, 659278.0347) | 289510.3826 (394659.444, 196505.6598) | 140.8123 (190.2581, 96.9758) | -6.3164 (-6.1245, -6.5080) |
| Greece | 8739.6409 (13683.8517, 4751.9734) | 4953.7173 (6275.7071, 3712.4105) | 16.1591 (20.2772, 12.2231) | -4.7432 (-4.2827, -5.2015) | 154050.8418 (239748.3714, 83761.0767) | 75587.9935 (94818.3905, 57372.0072) | 306.1395 (381.7916, 232.2126) | -4.3596 (-3.9640, -4.7535) |
| Iceland | 34.0907 (78.9385, 2.6591) | 11.4965 (25.7013, 2.1105) | 1.6739 (3.7304, 0.3082) | -6.4460 (-5.9857, -6.9042) | 599.9083 (1382.5703, 46.8657) | 178.7451 (393.4005, 33.1354) | 29.3566 (64.3289, 5.4289) | -6.6605 (-6.2623, -7.0569) |
| Ireland | 1552.3432 (2695.8929, 621.4135) | 328.8367 (511.2385, 163.1854) | 3.8740 (6.0235, 1.9283) | -7.5305 (-7.2306, -7.8295) | 29379.033 (50919.8828, 11787.2339) | 5469.0431 (8489.3543, 2768.4295) | 68.1265 (105.7396, 34.5012) | -7.7590 (-7.4744, -8.0428) |
| Israel | 2383.6112 (3615.0278, 1360.649) | 1350.73 (1705.5013, 1004.2347) | 9.6631 (12.1599, 7.2442) | -6.0254 (-5.8351, -6.2153) | 45215.6693 (68040.6558, 25947.7703) | 22637.4858 (28482.6254, 17459.2032) | 176.4695 (221.0980, 136.0573) | -5.9898 (-5.8125, -6.1668) |
| Italy | 39005.442 (57837.5321, 22344.4829) | 17987.8727 (23317.7387, 13048.3905) | 9.1766 (11.7639, 6.6601) | -5.2700 (-5.0362, -5.5033) | 690062.4508 (1011173.3034, 404688.8845) | 252109.7879 (323148.7591, 186855.5511) | 153.8687 (195.3999, 114.6355) | -5.4568 (-5.2481, -5.6650) |
| Luxembourg | 221.2281 (368.2524, 96.4732) | 50.3475 (74.0587, 28.0656) | 4.1417 (6.0810, 2.3032) | -7.1815 (-6.8470, -7.5149) | 3944.0851 (6531.4156, 1704.5123) | 801.6502 (1169.0695, 447.6849) | 71.7096 (104.6080, 40.4733) | -7.3530 (-7.0335, -7.6714) |
| Malta | 193.7062 (314.94, 90.1092) | 115.9167 (154.162, 79.3474) | 10.4604 (13.8755, 7.1941) | -5.0105 (-4.5914, -5.4277) | 3683.1386 (5991.9007, 1710.0031) | 1858.1242 (2467.6116, 1291.1242) | 187.9363 (249.0474, 130.8298) | -5.0699 (-4.6927, -5.4457) |
| Netherlands | 8561.3483 (13435.753, 4348.5296) | 2441.1427 (3248.4193, 1691.7333) | 6.1191 (8.1377, 4.2458) | -6.7106 (-6.4742, -6.9464) | 160443.0793 (249287.8475, 81721.2522) | 38379.839 (51137.2436, 27038.1011) | 104.9250 (139.6553, 73.7513) | -7.0421 (-6.7814, -7.3022) |
| Norway | 1836.6335 (3359.7292, 556.5704) | 231.9755 (408.1411, 85.4567) | 1.9357 (3.3921, 0.7157) | -8.7183 (-8.3328, -9.1021) | 32270.1729 (58550.6383, 9769.9872) | 3711.4832 (6517.2197, 1376.2032) | 34.8116 (61.2813, 12.9714) | -8.8541 (-8.5309, -9.1761) |
| Portugal | 4828.9288 (8416.6184, 1821.1069) | 1193.7975 (1834.4296, 642.7554) | 3.9396 (6.0127, 2.1534) | -7.8031 (-7.3489, -8.2551) | 86702.6575 (151356.3357, 32944.7309) | 18480.4643 (28245.7278, 10041.2193) | 72.3919 (109.9816, 39.8361) | -7.5109 (-7.0828, -7.9370) |
| Spain | 14640.663 (25386.938, 6471.6888) | 5061.301 (7128.7878, 3166.9226) | 4.0401 (5.6402, 2.5501) | -6.1825 (-5.8382, -6.5256) | 258061.6606 (441225.2607, 113803.3444) | 80797.2098 (114027.8671, 50822.2752) | 77.9411 (109.4130, 49.3907) | -5.9420 (-5.6106, -6.2722) |
| Sweden | 3768.9616 (7167.8948, 986.2004) | 587.7688 (1108.5992, 172.3188) | 2.1526 (4.0554, 0.6318) | -7.8731 (-7.4172, -8.3266) | 62399.7871 (118382.0663, 16140.0995) | 8841.1869 (16435.517, 2544.6497) | 37.1312 (69.3878, 10.6427) | -7.9294 (-7.5223, -8.3348) |
| Switzerland | 3967.36 (6494.6036, 1895.8764) | 978.1131 (1389.6302, 602.0204) | 4.0908 (5.7919, 2.5251) | -6.9259 (-6.6811, -7.1701) | 65824.8873 (106918.7547, 31643.653) | 13948.4388 (19684.1952, 8745.4025) | 68.0471 (95.8992, 42.9593) | -7.1031 (-6.8758, -7.3299) |
| United Kingdom | 42989.447 (70710.2621, 19765.7077) | 8561.4906 (11825.7991, 5416.95) | 5.7483 (7.9712, 3.6325) | -7.2872 (-7.0039, -7.5697) | 783706.7286 (1274687.1348, 358623.2664) | 142815.0705 (199711.0386, 91736.984) | 108.8519 (152.6336, 69.7903) | -7.3114 (-7.0477, -7.5743) |
| Argentina | 14612.3711 (23332.9596, 7033.766) | 7255.6566 (11566.6622, 3720.7978) | 12.5571 (19.9937, 6.4425) | -4.2883 (-3.9860, -4.5897) | 310614.1379 (493110.9982, 151350.2158) | 143077.5471 (226917.5116, 74084.9005) | 255.9207 (405.4527, 132.7236) | -4.3602 (-4.0717, -4.6477) |
| Chile | 5611.061 (7274.1896, 3959.481) | 4001.5362 (5433.5875, 2670.657) | 15.2851 (20.7578, 10.1921) | -4.4302 (-4.2648, -4.5953) | 116635.2995 (150666.0079, 82129.954) | 80539.5657 (108294.9541, 53304.6506) | 315.7187 (424.0916, 208.9159) | -4.2016 (-4.0381, -4.3648) |
| Uruguay | 1410.0181 (2399.1399, 557.9538) | 570.3332 (981.727, 217.8223) | 8.9522 (15.4652, 3.4152) | -4.8812 (-4.6315, -5.1303) | 27652.6593 (47142.8852, 10829.6719) | 9957.5824 (17128.2652, 3848.735) | 178.2190 (307.1658, 69.1835) | -4.9010 (-4.6327, -5.1684) |
| Canada | 5598.1955 (10721.2943, 1592.1703) | 1808.2044 (3243.9615, 657.0825) | 2.2134 (3.9719, 0.8018) | -7.0383 (-6.6261, -7.4487) | 104906.5774 (200299.1244, 29443.3177) | 30790.2836 (54595.1958, 11162.554) | 42.1269 (74.6921, 15.2787) | -6.8997 (-6.5331, -7.2649) |
| United States of America | 93887.4708 (164939.1871, 38656.304) | 29695.8149 (48063.0819, 14342.2624) | 4.7405 (7.6714, 2.2893) | -6.3324 (-5.9125, -6.7506) | 1738456.318 (3025802.687, 722361.1362) | 544374.1773 (875255.7423, 262642.1556) | 95.0585 (152.5317, 45.7976) | -6.1817 (-5.7899, -6.5719) |
| Antigua and Barbuda | 22.9702 (48.7283, 5.5649) | 21.5302 (38.3511, 7.7832) | 23.1194 (41.0433, 8.3289) | -1.9628 (-1.6868, -2.2380) | 435.1479 (922.7159, 104.573) | 437.627 (779.2408, 158.0389) | 427.6894 (757.2212, 154.0519) | -2.2334 (-1.9552, -2.5109) |
| Bahamas | 50.3218 (106.2638, 9.4036) | 77.2018 (147.137, 27.8062) | 21.1712 (40.4273, 7.6133) | -1.8008 (-1.5775, -2.0235) | 1245.3145 (2622.4325, 232.3011) | 1832.3089 (3504.3402, 666.5973) | 450.3609 (859.2197, 163.5935) | -1.8778 (-1.6664, -2.0887) |
| Barbados | 136.6843 (270.5312, 36.5231) | 135.0436 (230.1166, 55.5818) | 26.0136 (44.3556, 10.7140) | -1.8827 (-1.5068, -2.2572) | 2415.7127 (4747.7585, 641.5358) | 2446.8462 (4178.442, 1013.9462) | 478.5597 (818.5236, 198.4249) | -1.8964 (-1.5359, -2.2557) |
| Belize | 51.6473 (76.8553, 29.865) | 79.1623 (127.9622, 39.2662) | 29.7301 (47.9176, 14.7463) | -2.5126 (-2.0988, -2.9246) | 1112.2778 (1660.1083, 643.0066) | 1808.4816 (2902.4227, 906.4198) | 606.0723 (974.4104, 302.4488) | -2.6130 (-2.2265, -2.9980) |
| Cuba | 5536.8491 (9976.0824, 2200.1247) | 6131.2454 (9960.2535, 3042.7418) | 29.3376 (47.8138, 14.5312) | -2.3846 (-2.0974, -2.6711) | 110024.7442 (197718.9078, 43797.1238) | 112150.1777 (182714.55, 55078.2943) | 568.0419 (926.9811, 278.4956) | -2.2954 (-2.0038, -2.5863) |
| Dominica | 49.2488 (67.0591, 30.3134) | 26.3142 (43.3903, 11.8642) | 34.1816 (56.5007, 15.3625) | -2.8953 (-2.4457, -3.3429) | 926.0753 (1271.618, 573.3921) | 520.008 (863.8826, 235.974) | 638.7565 (1056.3406, 289.6760) | -2.8044 (-2.3303, -3.2761) |
| Dominican Republic | 2447.5752 (3216.1578, 1671.635) | 3787.4012 (6616.4164, 1438.662) | 38.9077 (67.7884, 14.7678) | -1.5877 (-1.2660, -1.9083) | 58962.7055 (77754.1587, 40177.8849) | 84909.0841 (147647.979, 31868.7605) | 840.8996 (1460.8971, 316.4039) | -1.4731 (-1.1257, -1.8193) |
| Grenada | 65.1022 (97.2898, 37.4552) | 37.0839 (65.3492, 14.805) | 37.4997 (65.9460, 15.0584) | -2.6367 (-2.3411, -2.9315) | 1299.335 (1933.7697, 743.3495) | 819.7379 (1452.8782, 322.696) | 741.2823 (1310.8044, 292.1984) | -2.8346 (-2.4747, -3.1931) |
| Guyana | 457.8977 (700.0337, 269.5236) | 356.9683 (582.064, 169.4224) | 63.8375 (104.0770, 30.0491) | -2.1255 (-1.9605, -2.2903) | 11539.2824 (17654.5929, 6749.4094) | 8658.0196 (14211.1347, 4140.5577) | 1354.6128 (2209.8414, 644.6989) | -2.2680 (-2.1437, -2.3920) |
| Haiti | 6580.3617 (7958.9772, 5342.0093) | 10673.1497 (14277.1888, 7803.1312) | 184.2158 (242.5222, 137.7048) | -0.8582 (-0.7962, -0.9201) | 172087.9118 (208948.7615, 139355.3432) | 276138.5036 (371632.4223, 201162.8635) | 3838.2212 (5116.2002, 2828.3755) | -0.9393 (-0.8651, -1.0133) |
| Jamaica | 1105.8124 (1451.9191, 708.1004) | 798.1832 (1205.1829, 438.9227) | 24.0297 (36.3977, 13.2616) | -2.3100 (-1.5436, -3.0705) | 21172.3235 (27781.5262, 13577.1764) | 14917.9994 (22641.6453, 8214.6876) | 472.0594 (717.7990, 259.9274) | -2.3142 (-1.5512, -3.0713) |
| Saint Lucia | 67.14 (101.1844, 36.6003) | 58.9498 (98.4391, 25.1993) | 25.7359 (42.9519, 11.0133) | -4.6304 (-4.0788, -5.1789) | 1339.4227 (2032.1122, 725.2743) | 1124.2858 (1897.7846, 475.6827) | 476.2150 (803.4334, 201.4356) | -4.3108 (-3.8161, -4.8030) |
| Saint Vincent and the Grenadines | 57.1964 (87.1962, 34.1507) | 48.1843 (85.6899, 19.4088) | 37.9786 (67.2608, 15.3084) | -2.6604 (-2.3035, -3.0161) | 1158.2484 (1779.1877, 693.6576) | 956.2546 (1684.9554, 382.9441) | 701.6912 (1239.0095, 281.3220) | -2.7751 (-2.4312, -3.1179) |
| Suriname | 206.2406 (320.4684, 104.9184) | 272.3441 (446.7047, 126.6811) | 45.0085 (73.8116, 20.9979) | -2.3897 (-2.0426, -2.7356) | 4833.1384 (7548.2792, 2416.0554) | 6350.2301 (10278.8573, 2965.5106) | 996.6290 (1611.8385, 466.7638) | -2.3997 (-2.0361, -2.7619) |
| Trinidad and Tobago | 521.7974 (1046.4173, 94.8998) | 685.9232 (1251.32, 246.887) | 36.8079 (67.0961, 13.2564) | -2.6022 (-2.2705, -2.9328) | 11718.6078 (23583.7529, 2137.6436) | 14642.015 (26654.043, 5247.947) | 771.4591 (1403.1443, 276.5716) | -2.5128 (-2.1571, -2.8671) |
| Bolivia (Plurinational State of) | 3219.5846 (4351.4092, 2374.2058) | 3041.7562 (4792.8654, 2010.4059) | 39.3863 (60.6652, 26.1643) | -3.7386 (-3.6548, -3.8224) | 79615.341 (108911.0304, 58353.5066) | 67930.5073 (105372.1748, 44366.1568) | 770.1474 (1193.8435, 509.2890) | -4.0411 (-3.9479, -4.1342) |
| Ecuador | 2980.7927 (3775.2428, 2095.8808) | 2988.2447 (4473.8223, 1814.8226) | 20.0991 (29.9057, 12.2820) | -4.2460 (-3.7175, -4.7717) | 69558.1875 (88025.4451, 48829.2918) | 61170.4373 (91316.069, 36496.7504) | 382.7377 (569.4795, 228.7185) | -4.4985 (-3.9809, -5.0133) |
| Peru | 6975.4432 (8468.739, 5671.6923) | 6728.3149 (9346.9252, 4644.0785) | 19.9962 (27.7571, 13.8163) | -4.3487 (-4.0143, -4.6819) | 163060.1604 (198980.1339, 131349.8488) | 145108.7989 (201749.8468, 101341.8561) | 424.2330 (589.6563, 295.8513) | -4.2950 (-3.9878, -4.6012) |
| Colombia | 10161.9197 (13161.588, 6890.9939) | 8806.707 (12732.4027, 5642.5442) | 15.5616 (22.5196, 9.9723) | -4.9763 (-4.8644, -5.0880) | 238671.9429 (310184.6786, 160248.5647) | 169172.4666 (244237.8666, 108484.7008) | 304.2254 (439.6513, 195.1085) | -5.1691 (-5.0565, -5.2816) |
| Costa Rica | 725.2769 (998.9337, 431.1883) | 573.3695 (813.1213, 374.024) | 10.1764 (14.4426, 6.6586) | -4.6175 (-4.3707, -4.8636) | 14860.8229 (20493.6142, 8717.5832) | 11735.5681 (16508.4479, 7682.1761) | 211.8793 (297.8162, 138.4656) | -4.4348 (-4.2004, -4.6687) |
| El Salvador | 2114.7591 (2454.4732, 1711.7927) | 1787.9161 (2531.1702, 1089.6527) | 26.6249 (37.9657, 16.2886) | -3.3696 (-3.1952, -3.5437) | 48461.6975 (56184.2693, 40181.3875) | 34608.9153 (49657.7705, 21324.2991) | 546.0041 (785.2042, 336.9307) | -3.5508 (-3.3714, -3.7299) |
| Guatemala | 2380.2868 (2742.0608, 1956.9262) | 4253.4459 (5567.5657, 2868.8277) | 46.0636 (59.8701, 30.5106) | -2.3754 (-2.0968, -2.6532) | 60623.3377 (69853.2381, 49850.4734) | 89523.1645 (117947.2659, 60184.7562) | 844.0794 (1105.6659, 569.0582) | -2.6679 (-2.3593, -2.9755) |
| Honduras | 1560.6826 (1847.6169, 1242.583) | 5021.2621 (6462.1626, 3776.2222) | 96.7109 (124.3105, 72.6763) | 0.4671 (0.7515, 0.1835) | 39197.2264 (46296.8702, 31742.8727) | 110017.0757 (140575.0157, 82172.7559) | 1825.6377 (2315.5999, 1376.2639) | 0.1202 (0.3439, -0.1029) |
| Mexico | 17457.4624 (23367.9685, 11493.6502) | 25007.4227 (35462.2105, 15930.8126) | 21.9106 (31.0777, 13.9528) | -3.0098 (-2.7782, -3.2408) | 381714.4981 (510164.4122, 249552.3758) | 513773.0426 (733702.307, 328415.8528) | 415.9076 (594.3744, 265.7043) | -2.9531 (-2.7373, -3.1683) |
| Nicaragua | 858.7748 (1003.6412, 721.6366) | 1506.4583 (1957.296, 1108.29) | 35.8285 (46.1877, 26.3842) | -1.6308 (-1.3455, -1.9152) | 20080.6131 (23163.3859, 16974.9636) | 32935.5117 (42513.6689, 24509.3569) | 692.0105 (893.0032, 507.8429) | -1.8820 (-1.6366, -2.1269) |
| Panama | 623.9666 (862.643, 365.987) | 450.5102 (732.8923, 243.179) | 9.8402 (15.9647, 5.3026) | -5.0338 (-4.8224, -5.2448) | 12918.4425 (17795.252, 7583.4151) | 8830.557 (14340.2711, 4690.6969) | 197.5722 (320.5079, 104.7736) | -4.9346 (-4.7390, -5.1299) |
| Venezuela (Bolivarian Republic of) | 4329.6683 (6953.5463, 2162.5373) | 7576.3942 (11805.2356, 4210.2216) | 26.7084 (41.5188, 14.8101) | -2.3364 (-2.1818, -2.4907) | 103388.1537 (165163.1055, 51850.5341) | 165181.0821 (260678.2981, 92064.2474) | 554.1965 (871.6530, 308.5544) | -2.4638 (-2.3044, -2.6230) |
| Brazil | 46740.4286 (68309.3731, 27435.7109) | 33455.0929 (48069.2651, 19573.4434) | 13.6584 (19.6412, 7.9680) | -4.8467 (-4.7059, -4.9872) | 1158956.878 (1699945.3609, 671653.4134) | 770091.2925 (1106566.483, 450520.3553) | 305.5272 (438.7638, 178.9598) | -4.7696 (-4.6188, -4.9202) |
| Paraguay | 1570.147 (2034.8577, 986.1348) | 1324.8396 (2409.14, 436.0333) | 24.2970 (44.0843, 7.9649) | -3.5704 (-3.3243, -3.8159) | 34534.7042 (45054.3616, 22031.8862) | 28671.2934 (52178.7907, 9488.0794) | 494.7849 (899.8803, 164.0100) | -3.6783 (-3.3829, -3.9729) |
| Algeria | 7584.3468 (10184.6585, 5174.6511) | 17096.0809 (23373.7626, 11217.1104) | 68.7873 (93.2150, 46.2354) | -1.2797 (-1.0867, -1.4723) | 179039.7806 (240658.5355, 121356.1486) | 349133.4619 (480608.6147, 231377.2945) | 1119.9920 (1525.5738, 743.4732) | -1.7077 (-1.4765, -1.9383) |
| Bahrain | 261.5765 (311.8962, 207.9182) | 464.884 (579.5665, 361.1034) | 95.1705 (116.4740, 74.6441) | -3.3845 (-2.9546, -3.8126) | 7081.9189 (8407.3521, 5646.9015) | 12662.724 (15848.2061, 9836.0921) | 1618.2532 (1987.1595, 1269.3713) | -3.7894 (-3.4051, -4.1723) |
| Egypt | 47011.918 (56903.5647, 36932.5717) | 93469.5346 (117999.2535, 71594.8973) | 208.9764 (259.4060, 160.8278) | -0.1147 (0.1320, -0.3608) | 1191516.6616 (1442619.1468, 940318.0891) | 2359204.3597 (2974191.4805, 1798635.0638) | 4028.0615 (5048.7465, 3098.6816) | -0.2136 (0.0101, -0.4368) |
| Iran (Islamic Republic of) | 20934.8152 (25172.8129, 16779.7273) | 38939.7375 (46895.196, 30705.7605) | 58.2497 (70.1062, 45.6383) | -2.1328 (-2.0248, -2.2406) | 523904.9534 (631501.7106, 421329.8093) | 814713.0148 (969581.9765, 653102.3523) | 1085.1715 (1295.1876, 871.0817) | -2.2709 (-2.1690, -2.3728) |
| Iraq | 11041.4356 (14276.6432, 8161.6084) | 23193.0703 (30735.5902, 16301.2802) | 126.6120 (166.2002, 90.8291) | -0.8294 (-0.6726, -0.9859) | 263036.8745 (341857.5363, 191061.6679) | 548455.9115 (748473.008, 380361.678) | 2428.5555 (3215.1441, 1705.3618) | -1.2173 (-1.0726, -1.3617) |
| Jordan | 756.0398 (961.1479, 559.1639) | 2428.8613 (3187.9521, 1753.4017) | 43.8885 (57.3135, 32.1822) | -2.0955 (-1.7585, -2.4314) | 19642.891 (25105.2221, 14556.9249) | 61368.6947 (80478.6341, 44334.2356) | 856.5456 (1110.9491, 629.9808) | -2.3002 (-1.9668, -2.6325) |
| Kuwait | 478.6842 (568.3714, 376.5608) | 1365.2269 (1782.4926, 1004.7121) | 53.3763 (69.3310, 39.6490) | -1.5274 (-0.9128, -2.1382) | 14064.0401 (16677.363, 11007.7888) | 37895.3901 (48969.5154, 28105.4002) | 1116.1646 (1440.1118, 829.9152) | -1.6036 (-1.0182, -2.1856) |
| Lebanon | 1097.5911 (1608.1849, 679.343) | 1680.5167 (2411.2618, 1084.3409) | 25.4873 (36.5681, 16.4446) | -2.8961 (-2.5714, -3.2197) | 25922.998 (38146.581, 15881.8427) | 30324.6127 (43339.538, 19375.9812) | 484.7730 (691.4292, 308.1880) | -3.1024 (-2.7271, -3.4763) |
| Libya | 1107.832 (1500.3592, 782.8852) | 3351.1674 (4593.0584, 2325.8161) | 74.3996 (102.3484, 52.1648) | 0.9393 (1.3610, 0.5193) | 26326.7532 (35552.8094, 18818.0269) | 84297.9284 (116779.816, 59321.7896) | 1549.7296 (2141.8640, 1081.3299) | 0.7887 (1.1774, 0.4014) |
| Morocco | 14257.891 (18103.3106, 10646.23) | 22646.8986 (29745.7835, 15827.6747) | 77.3702 (100.7701, 54.1477) | -1.0517 (-0.8364, -1.2665) | 335896.9156 (425713.5468, 246506.8332) | 496309.1339 (662620.5983, 346753.123) | 1498.2624 (1987.2542, 1053.2220) | -1.3513 (-1.1270, -1.5750) |
| Palestine | 832.763 (1122.8729, 582.9125) | 1403.2735 (1827.8567, 957.99) | 77.1779 (100.2340, 53.1284) | -1.4413 (-1.0925, -1.7889) | 17656.9649 (24118.9838, 12115.3775) | 31819.1573 (41575.0651, 21767.076) | 1375.0546 (1784.7667, 943.2021) | -1.5726 (-1.2665, -1.8777) |
| Oman | 856.5999 (1166.6126, 597.6852) | 1206.8857 (1553.8754, 845.3536) | 85.2060 (109.5421, 60.8069) | -1.3332 (-1.1576, -1.5084) | 22262.2408 (30996.2463, 15357.5226) | 32126.9725 (41425.3321, 22141.601) | 1616.7366 (2067.7694, 1135.3169) | -1.8127 (-1.6289, -1.9962) |
| Qatar | 152.7664 (187.3233, 118.9424) | 386.006 (524.4951, 272.4639) | 76.5255 (98.6019, 55.8202) | -4.3884 (-3.6203, -5.1503) | 4535.017 (5604.1867, 3548.8543) | 12903.9901 (17237.4942, 9155.8506) | 1334.9401 (1748.3379, 968.0807) | -4.3401 (-3.6690, -5.0065) |
| Saudi Arabia | 6356.0418 (8445.5359, 4540.163) | 17417.5646 (22425.6526, 12686.8681) | 108.3596 (135.1995, 82.6149) | -0.5959 (-0.3514, -0.8398) | 164958.959 (222347.9946, 115930.2671) | 571456.8712 (746144.5155, 407940.3941) | 2357.9948 (2965.3217, 1752.3638) | -0.3357 (-0.0774, -0.5934) |
| Syrian Arab Republic | 5594.4779 (7557.8105, 3899.9985) | 10984.6741 (15222.4969, 7433.2639) | 109.8995 (149.4290, 76.1267) | -0.5613 (-0.3749, -0.7474) | 140550.7696 (191550.5105, 96775.158) | 255111.3342 (358293.253, 170956.4197) | 2071.7420 (2866.9920, 1404.1424) | -0.9350 (-0.7313, -1.1383) |
| Tunisia | 3066.1704 (4076.5624, 2204.243) | 6104.2121 (8735.3021, 4009.5662) | 52.0998 (74.2826, 34.3125) | -1.5025 (-1.2654, -1.7389) | 69366.3025 (92335.5217, 49690.8485) | 122313.9058 (174491.2287, 80695.3206) | 959.3440 (1358.5252, 635.2005) | -1.5790 (-1.3428, -1.8146) |
| Turkey | 23251.9121 (30485.3171, 16945.1471) | 36490.2363 (45925.0482, 27403.15) | 43.6742 (54.8425, 32.6723) | -1.6786 (-1.2709, -2.0845) | 550771.5087 (718051.4072, 401953.0718) | 710848.4616 (896093.3432, 523464.5265) | 790.0876 (992.7786, 583.3829) | -2.2730 (-1.9433, -2.6015) |
| United Arab Emirates | 440.4995 (576.1243, 324.8023) | 1343.6676 (1767.2129, 951.8871) | 83.1838 (106.1851, 61.2863) | 0.2040 (0.8318, -0.4200) | 13472.6295 (17847.6352, 9891.3736) | 46364.1347 (60118.6447, 33063.6395) | 1463.4579 (1858.5294, 1070.5401) | -0.7593 (-0.2459, -1.2701) |
| Yemen | 9222.5854 (12019.3749, 6789.5907) | 17428.114 (23996.5793, 12226.6672) | 153.4705 (208.0587, 107.6818) | -1.5255 (-1.4161, -1.6347) | 246831.3405 (324963.8738, 177887.6401) | 439684.0991 (608801.5072, 306048.9875) | 3113.7598 (4279.1035, 2193.1777) | -1.7625 (-1.6586, -1.8663) |
| Afghanistan | 17407.9121 (21706.6376, 13076.6225) | 17075.466 (21822.0815, 13101.856) | 208.9015 (261.4788, 162.9161) | -1.2480 (-1.0904, -1.4054) | 445283.0117 (561343.9059, 328198.3068) | 478413.0449 (631731.8552, 359175.2613) | 4510.8856 (5737.1083, 3510.7571) | -1.4382 (-1.2684, -1.6076) |
| Bangladesh | 67342.0585 (80019.5065, 56262.9685) | 142723.3653 (178527.1566, 109188.0768) | 117.7515 (145.9221, 90.6931) | -0.8846 (-0.6170, -1.1514) | 1763463.8903 (2100937.7817, 1467800.4097) | 3258478.7824 (4137725.0333, 2460822.9247) | 2400.4441 (3023.0086, 1821.9295) | -1.1555 (-0.9905, -1.3202) |
| Bhutan | 193.6039 (248.7231, 140.9525) | 294.102 (382.0501, 208.2963) | 52.6397 (68.0802, 37.5600) | -2.1347 (-1.9633, -2.3057) | 5419.7588 (7017.2868, 3941.4208) | 6540.1358 (8660.0898, 4600.5582) | 1080.8536 (1420.7495, 763.5137) | -2.4898 (-2.3146, -2.6646) |
| India | 428415.6932 (505046.982, 356058.1046) | 964920.3612 (1148717.3844, 799550.6653) | 89.2817 (105.5707, 73.6045) | -0.4530 (-0.2875, -0.6183) | 12133364.2656 (14252232.6544, 10123305.0148) | 24228447.0244 (28896099.3896, 20105480.0408) | 2006.3881 (2385.2870, 1664.0329) | -0.6588 (-0.5422, -0.7752) |
| Nepal | 9613.0897 (11949.5079, 7452.8487) | 20151.6449 (25582.6114, 15274.2453) | 100.4017 (126.2418, 76.6951) | -0.4021 (-0.2093, -0.5945) | 261201.9244 (323999.8939, 202626.1464) | 478904.6073 (611916.8158, 362133.6719) | 2092.8937 (2656.1850, 1582.4584) | -0.6682 (-0.4756, -0.8604) |
| Pakistan | 57455.3848 (68584.6424, 44483.8453) | 122214.9881 (153287.6362, 95773.0099) | 118.3504 (148.1422, 92.4633) | -0.0568 (0.1285, -0.2418) | 1439820.4409 (1715999.7541, 1121082.8635) | 3291302.2939 (4139101.8654, 2571994.8863) | 2593.1266 (3243.5345, 2040.9441) | -0.1176 (0.0887, -0.3235) |
| Angola | 4246.5242 (5430.6586, 3329.7282) | 6983.5183 (9438.8022, 4596.125) | 81.1427 (109.5948, 55.5353) | -2.2487 (-2.0761, -2.4211) | 117200.3014 (149910.5582, 90893.1716) | 183981.0861 (251459.8047, 120018.023) | 1658.0742 (2225.1289, 1112.9306) | -2.4546 (-2.2853, -2.6236) |
| Central African Republic | 1690.79 (2244.8788, 1309.9052) | 2745.5524 (3744.6812, 1951.2519) | 166.3625 (219.2525, 123.3461) | -0.5016 (-0.4379, -0.5652) | 47090.2077 (63272.1175, 36330.4919) | 78911.7518 (110449.4149, 55794.9001) | 3603.5872 (4847.3428, 2627.9793) | -0.5894 (-0.5173, -0.6613) |
| Congo | 1561.1207 (1921.2271, 1203.3237) | 2291.1341 (2980.6943, 1677.2204) | 113.5175 (142.3845, 84.7032) | -1.9220 (-1.7531, -2.0906) | 41162.8112 (52400.051, 31353.4548) | 60324.238 (80706.6108, 43265.1077) | 2323.9607 (2998.2185, 1713.9529) | -2.1387 (-1.9566, -2.3204) |
| Democratic Republic of the Congo | 16127.5786 (20696.0603, 12358.7542) | 32587.0927 (42698.1757, 24127.308) | 118.8622 (154.6945, 89.2108) | -0.5665 (-0.5280, -0.6050) | 425963.0571 (545366.352, 324871.7823) | 841931.0594 (1113027.225, 625153.0668) | 2437.6038 (3181.4837, 1820.5656) | -0.6398 (-0.5999, -0.6798) |
| Equatorial Guinea | 272.444 (340.2035, 207.1926) | 247.4004 (405.468, 131.3513) | 64.9340 (104.4058, 35.3572) | -3.8184 (-3.1179, -4.5138) | 7202.9669 (9067.9225, 5482.2092) | 6178.1966 (10326.6662, 3210.6768) | 1281.1649 (2081.1214, 686.6784) | -4.2142 (-3.4804, -4.9424) |
| Gabon | 385.6212 (576.255, 216.0256) | 468.3498 (707.959, 280.2322) | 57.7367 (85.7214, 34.0817) | -0.9725 (-0.8243, -1.1205) | 8948.7852 (13486.1823, 4974.1177) | 11312.3773 (17314.1291, 6736.0159) | 1150.5566 (1739.9938, 690.6629) | -1.1169 (-0.9713, -1.2624) |
| Burundi | 3681.1174 (4679.7991, 2750.5913) | 4284.3004 (5367.8737, 3331.8163) | 109.9592 (138.6430, 86.0930) | -2.0753 (-1.8394, -2.3107) | 95518.2676 (122211.3511, 70961.7037) | 115734.2137 (145289.8623, 89621.3424) | 2356.5567 (2925.3453, 1840.9389) | -2.2693 (-2.0139, -2.5239) |
| Comoros | 200.9981 (251.4176, 150.9695) | 330.8077 (430.5425, 249.2187) | 80.4811 (104.6552, 61.2708) | -1.7082 (-1.4710, -1.9448) | 5344.5136 (6767.4361, 3968.7622) | 8039.5052 (10367.996, 5967.2105) | 1686.1038 (2174.5232, 1270.0797) | -1.8960 (-1.6351, -2.1562) |
| Djibouti | 94.0844 (127.1633, 65.0956) | 339.8455 (493.1675, 216.7185) | 71.4120 (101.5595, 45.9993) | -0.8491 (-0.7278, -0.9701) | 2741.696 (3710.9829, 1872.0699) | 9534.2702 (13851.6604, 6107.0074) | 1512.4582 (2166.4702, 978.3977) | -0.9523 (-0.8340, -1.0705) |
| Eritrea | 1338.2668 (1703.3359, 1020.5461) | 2302.5474 (3013.2322, 1677.2211) | 108.5166 (138.9726, 81.3550) | -1.0104 (-0.9269, -1.0938) | 42042.5078 (53807.6011, 31754.5524) | 66126.398 (87617.0395, 47724.3182) | 2347.6193 (3042.1907, 1741.3939) | -1.2646 (-1.1680, -1.3612) |
| Ethiopia | 20402.9025 (26798.5699, 16297.996) | 23607.3727 (28705.2389, 18603.544) | 64.0707 (78.1060, 50.1699) | -2.4433 (-2.3203, -2.5661) | 580375.0828 (773787.4618, 460806.1097) | 591867.4204 (721286.5501, 470355.1071) | 1358.7548 (1653.2710, 1079.2226) | -2.7937 (-2.6528, -2.9344) |
| Kenya | 4446.4804 (5606.8458, 3342.9283) | 12471.5689 (16038.5277, 9523.1799) | 72.5461 (94.2301, 55.1268) | 0.5932 (0.7805, 0.4063) | 109336.4953 (137037.9628, 83797.2991) | 311888.1622 (396960.8306, 242243.649) | 1452.5802 (1849.7596, 1127.6796) | 0.4531 (0.6890, 0.2178) |
| Madagascar | 7186.7672 (8499.2437, 6015.0646) | 13099.1337 (17130.9453, 9762.5983) | 147.6125 (190.8167, 110.9566) | -0.4674 (-0.4072, -0.5276) | 194299.7315 (229178.4406, 162306.8373) | 383222.5105 (505303.1847, 286043.6111) | 3268.4796 (4224.5645, 2468.2997) | -0.5586 (-0.4995, -0.6176) |
| Malawi | 3541.3546 (4166.9264, 2826.1236) | 7008.5689 (8616.4583, 5575.744) | 116.1210 (142.6192, 93.0579) | -0.1859 (0.0330, -0.4042) | 93827.0715 (110754.751, 74989.0573) | 185629.6187 (226403.9464, 145101.561) | 2494.8429 (3037.8389, 1991.7974) | -0.2617 (-0.0173, -0.5054) |
| Mauritius | 240.114 (383.8359, 110.508) | 188.9324 (334.9815, 64.7214) | 11.1003 (19.6069, 3.7956) | -4.5947 (-4.1233, -5.0637) | 6148.5407 (9892.1303, 2823.2194) | 4295.0186 (7652.3843, 1497.2249) | 244.5813 (435.8394, 85.1886) | -4.5237 (-4.0620, -4.9832) |
| Mozambique | 5935.9102 (7137.5424, 4785.7426) | 12334.9615 (15584.163, 9116.92) | 132.7362 (167.6160, 99.6203) | 0.8156 (0.9959, 0.6356) | 154253.2117 (185227.9982, 123933.6408) | 338471.1208 (432644.8189, 247595.8849) | 2981.5765 (3769.3920, 2212.2942) | 0.9241 (1.1264, 0.7222) |
| Rwanda | 4605.5909 (5746.037, 3656.3208) | 4639.7441 (6071.5516, 3377.4607) | 95.3285 (125.5875, 69.9814) | -3.2912 (-2.8825, -3.6981) | 126477.8492 (159595.1103, 99162.5531) | 116957.2198 (154901.2503, 85161.5006) | 1938.8210 (2523.7979, 1436.5212) | -3.7344 (-3.2939, -4.1730) |
| Seychelles | 10.0434 (17.7351, 3.019) | 11.4724 (21.2431, 4.0325) | 11.1875 (20.7051, 3.9405) | -2.0018 (-1.7232, -2.2796) | 228.6654 (402.05, 68.4389) | 270.6409 (494.7296, 96.7262) | 235.9221 (430.5276, 84.3212) | -2.2764 (-1.9884, -2.5636) |
| Somalia | 2685.1671 (3568.1979, 1974.3687) | 5191.6184 (7227.0221, 3641.1397) | 107.9733 (146.6048, 77.5545) | -0.8131 (-0.7751, -0.8510) | 80398.5758 (109634.6918, 58141.7079) | 153869.7731 (216898.6193, 106508.9146) | 2428.3496 (3349.6723, 1732.4733) | -0.9256 (-0.8832, -0.9680) |
| United Republic of Tanzania | 8973.3739 (11080.3349, 7127.5926) | 20557.3604 (26465.8208, 15678.9613) | 97.7375 (124.7475, 75.3173) | -0.1126 (-0.0341, -0.1911) | 232440.7796 (284891.9042, 185275.6755) | 506200.2327 (654036.4247, 380307.93) | 2026.5536 (2600.3746, 1553.2857) | -0.3576 (-0.2803, -0.4348) |
| Uganda | 6025.7766 (7539.6054, 4714.6147) | 9935.2009 (12825.1883, 7499.9014) | 83.4900 (106.1974, 63.7702) | -1.5936 (-1.3186, -1.8679) | 151557.1849 (191681.4254, 118929.2065) | 258308.8246 (334303.3402, 194813.2382) | 1772.2990 (2262.4977, 1350.7746) | -1.6867 (-1.3909, -1.9816) |
| Zambia | 2707.9626 (3286.1808, 2161.9) | 6326.1916 (8183.1923, 4500.6196) | 117.0294 (149.1036, 85.3011) | -0.1933 (-0.0704, -0.3160) | 70674.8275 (85699.383, 56639.5815) | 166265.0459 (215289.9428, 117550.6) | 2438.0755 (3128.9163, 1753.5048) | -0.3568 (-0.2144, -0.4990) |
| Botswana | 480.1817 (634.5803, 326.5919) | 406.2585 (680.1316, 230.8313) | 36.0919 (61.0156, 20.6290) | -3.4443 (-3.1593, -3.7284) | 12260.2786 (16399.3299, 8243.7645) | 10026.7453 (16647.7856, 5625.8598) | 728.3198 (1217.3262, 414.1072) | -3.6002 (-3.3274, -3.8722) |
| Lesotho | 616.2594 (762.752, 469.6378) | 1031.8295 (1416.0076, 712.6903) | 116.8003 (154.8225, 83.3770) | 2.0092 (2.5497, 1.4716) | 13803.2853 (16977.8822, 10414.3741) | 25905.974 (36634.6692, 17479.9602) | 2480.0005 (3423.4379, 1715.3724) | 2.1309 (2.6863, 1.5785) |
| Namibia | 599.7742 (750.5148, 437.0826) | 714.6793 (1151.9771, 390.8005) | 66.3481 (104.7590, 36.3912) | -2.2028 (-1.7912, -2.6125) | 14977.7777 (18849.1471, 10835.1416) | 17057.595 (27833.9216, 9232.9227) | 1318.0783 (2120.7114, 721.6490) | -2.3353 (-1.9037, -2.7649) |
| South Africa | 8577.1454 (10927.6318, 6241.4947) | 14517.1646 (18700.6231, 10455.7298) | 37.6212 (48.7134, 26.9322) | -0.7974 (-0.2572, -1.3348) | 228900.6178 (289774.0108, 171674.3153) | 342275.5884 (445358.6201, 248519.1871) | 762.0346 (991.5964, 549.3509) | -1.1693 (-0.6734, -1.6627) |
| Eswatini | 258.1782 (325.581, 191.4568) | 356.8486 (567.6286, 191.1577) | 81.1937 (122.5299, 45.1891) | -0.6653 (-0.1148, -1.2128) | 6468.4014 (8241.5458, 4883.408) | 9481.1083 (15346.7935, 5014.8618) | 1728.9164 (2718.2571, 937.5876) | -0.6001 (0.0239, -1.2202) |
| Zimbabwe | 2662.3442 (3246.5048, 2108.4767) | 6151.3171 (7843.8375, 4693.5029) | 115.9944 (144.9624, 90.1198) | 1.7375 (2.2843, 1.1937) | 62536.2732 (76400.3772, 50608.2449) | 162299.6122 (211036.1212, 121558.9186) | 2433.7836 (3084.4139, 1866.1092) | 1.9544 (2.5467, 1.3656) |
| Benin | 2303.2766 (2744.5294, 1912.6345) | 4530.1674 (5567.3648, 3594.3191) | 109.4929 (133.1350, 88.4789) | -0.5395 (-0.4324, -0.6466) | 52457.7524 (62609.75, 43484.5434) | 107139.4333 (133707.1262, 83157.7232) | 2173.4567 (2672.1201, 1715.7865) | -0.6889 (-0.5654, -0.8122) |
| Burkina Faso | 3498.0743 (4192.571, 2818.7302) | 7385.6225 (9139.7247, 5786.9879) | 97.9756 (119.7836, 77.7982) | 0.2336 (0.3424, 0.1249) | 87930.9741 (105817.9793, 70732.8226) | 177657.4454 (224071.0361, 136319.0812) | 1986.6468 (2466.6153, 1558.5470) | 0.0403 (0.1177, -0.0370) |
| Cameroon | 3992.1333 (4939.9505, 3088.6148) | 11081.0802 (15180.7765, 8137.0886) | 112.0362 (150.1683, 83.8881) | 0.1022 (0.4983, -0.2923) | 101736.0634 (126433.6246, 78282.0681) | 285234.0667 (390815.3004, 207991.8907) | 2308.9673 (3133.6259, 1711.6712) | 0.0070 (0.4280, -0.4121) |
| Cabo Verde | 180.8432 (216.1701, 141.2616) | 318.1131 (416.4327, 230.0973) | 76.8837 (100.7830, 55.8253) | -0.5143 (0.0143, -1.0401) | 3770.1512 (4482.8102, 2995.8729) | 6404.2646 (8426.3075, 4647.227) | 1466.3766 (1927.9065, 1065.5645) | -0.9013 (-0.4276, -1.3727) |
| Chad | 2962.3606 (3572.0525, 2397.7887) | 6030.7013 (7826.1417, 4562.7826) | 128.4862 (163.6680, 98.3066) | 0.2013 (0.3922, 0.0107) | 70095.4425 (83928.085, 57030.8227) | 154602.6513 (202103.5125, 116371.753) | 2712.3037 (3500.9816, 2064.6399) | 0.1475 (0.3563, -0.0608) |
| C么te d'Ivoire | 3995.7005 (4939.119, 3173.0384) | 10200.4341 (13515.034, 7712.7065) | 118.4967 (153.5740, 91.0513) | -0.4359 (-0.2337, -0.6377) | 111655.7807 (138760.0453, 88359.3292) | 270203.816 (360634.5257, 199388.5647) | 2446.2834 (3196.4066, 1867.8162) | -0.5707 (-0.3550, -0.7859) |
| Gambia | 382.9153 (480.3928, 299.8052) | 1219.1581 (1522.8192, 924.6337) | 149.6208 (184.8633, 114.9117) | 0.2738 (0.3739, 0.1737) | 9912.3978 (12483.131, 7650.2464) | 29316.1446 (36855.0055, 22126.1176) | 3055.6715 (3806.9986, 2330.8860) | 0.1015 (0.2334, -0.0303) |
| Ghana | 8482.5106 (10442.7177, 6784.2963) | 16845.6645 (21201.1247, 12435.9722) | 126.9257 (158.1699, 96.4251) | -0.8941 (-0.7620, -1.0260) | 229218.6722 (282949.493, 182132.6799) | 438758.3792 (553676.2635, 325339.1437) | 2651.1003 (3322.5865, 1973.3984) | -1.0023 (-0.8606, -1.1439) |
| Guinea | 3604.4796 (4416.4422, 2840.521) | 6208.2113 (8019.3111, 4697.2918) | 129.8313 (165.4750, 100.6783) | 0.4247 (0.5591, 0.2905) | 85625.5713 (104954.5636, 68236.2844) | 149867.6476 (195168.9555, 111688.5477) | 2700.3277 (3477.0362, 2049.9523) | 0.3354 (0.4689, 0.2021) |
| Guinea-Bissau | 666.1363 (870.0738, 506.8051) | 1030.7763 (1303.0708, 782.4895) | 183.8848 (226.4897, 142.0107) | -0.1256 (-0.0633, -0.1878) | 18218.5944 (23960.6792, 13723.0751) | 29029.1121 (36941.8964, 21934.481) | 3943.7459 (4931.7391, 3026.1279) | -0.3427 (-0.2768, -0.4084) |
| Liberia | 1290.6467 (1518.7292, 1038.4497) | 2105.8452 (2762.3717, 1596.9989) | 126.1362 (164.6433, 97.2257) | -0.2502 (-0.1385, -0.3617) | 30964.8103 (36916.6466, 24789.0826) | 54642.4817 (72181.035, 41151.4485) | 2581.8157 (3357.9213, 1960.2450) | -0.3276 (-0.2078, -0.4473) |
| Mali | 3456.5559 (4188.2409, 2781.3382) | 6387.5647 (7983.6034, 4928.0253) | 91.0917 (112.6697, 71.5652) | -0.4134 (-0.3008, -0.5260) | 91139.175 (110119.7424, 73162.5291) | 164103.7143 (206481.9281, 127049.2136) | 1900.0485 (2367.7718, 1483.1656) | -0.5551 (-0.4402, -0.6699) |
| Mauritania | 1355.9155 (1663.8969, 1064.0576) | 1958.4153 (2544.223, 1450.25) | 108.5723 (140.8426, 81.6530) | -1.4000 (-1.2218, -1.5779) | 32605.536 (40202.5712, 25591.7582) | 44241.5212 (58559.3251, 32368.701) | 2137.6030 (2800.0276, 1583.7662) | -1.6344 (-1.4617, -1.8069) |
| Niger | 2325.5653 (2927.0573, 1754.6546) | 6516.0444 (8501.8439, 4813.1773) | 103.5324 (132.8116, 79.1080) | -0.0811 (-0.0042, -0.1579) | 62267.6695 (78426.7742, 46553.1352) | 163714.8532 (214726.2796, 118389.872) | 2098.1312 (2701.5127, 1577.5347) | -0.2411 (-0.1543, -0.3277) |
| Nigeria | 48512.6317 (60166.2073, 38495.8414) | 62903.2481 (79673.2073, 49923.7807) | 89.4765 (110.6697, 72.0616) | -1.4103 (-1.2630, -1.5574) | 1128096.2571 (1410680.3285, 893417.4299) | 1492554.4489 (1937396.859, 1159397.5703) | 1736.8735 (2201.8880, 1364.7259) | -1.5663 (-1.4065, -1.7259) |
| Sao Tome and Principe | 59.0125 (68.504, 48.2567) | 71.794 (88.8074, 55.8423) | 79.9281 (98.4550, 62.8038) | -0.8100 (-0.5548, -1.0646) | 1348.6043 (1583.0941, 1095.5423) | 1780.6004 (2233.2218, 1358.301) | 1630.8876 (2008.7385, 1271.8439) | -1.0097 (-0.7046, -1.3140) |
| Senegal | 3849.5101 (4610.5513, 3133.1165) | 7996.8234 (10288.8024, 6118.4734) | 125.3544 (160.9772, 97.4527) | -0.4331 (-0.3660, -0.5001) | 95091.242 (113980.3375, 77353.9382) | 187357.0513 (241233.5528, 143210.3524) | 2509.9277 (3216.4996, 1930.5661) | -0.6053 (-0.5291, -0.6815) |
| Sierra Leone | 2609.3383 (3138.0715, 2071.6073) | 4316.6997 (5499.8708, 3258.1225) | 137.0845 (171.6746, 106.6510) | 0.0276 (0.2154, -0.1598) | 61520.2852 (74534.6238, 48911.3619) | 107322.8968 (139594.448, 79085.9302) | 2852.9951 (3646.1736, 2145.3955) | 0.0179 (0.2127, -0.1765) |
| Togo | 1394.161 (1668.6778, 1135.1915) | 3931.8284 (5171.7903, 2922.6424) | 134.4909 (172.4491, 102.4317) | -0.2345 (-0.0782, -0.3905) | 36538.2631 (43695.2771, 29751.8586) | 104335.7061 (138255.525, 76202.8392) | 2788.4758 (3638.1158, 2093.6717) | -0.3090 (-0.1379, -0.4798) |
| American Samoa | 2.6762 (9.6059, 0) | 6.3862 (14.6603, 0.6796) | 15.3770 (34.9506, 1.6332) | -0.0322 (0.1704, -0.2344) | 77.5241 (277.6458, 0) | 165.3445 (382.5218, 17.512) | 345.6997 (798.7835, 36.6436) | -0.0409 (0.1687, -0.2500) |
| Bermuda | 12.3779 (32.6641, 0) | 6.3837 (12.2434, 1.1827) | 4.1944 (8.0229, 0.7757) | -5.9900 (-5.5630, -6.4150) | 251.1343 (663.4195, 0) | 109.3979 (207.9688, 20.3488) | 79.1729 (150.6587, 14.7156) | -5.9685 (-5.5352, -6.3997) |
| Cook Islands | 1.9807 (5.3781, 0.0726) | 1.4902 (3.6188, 0) | 5.9663 (14.4508, 0.0000) | -3.6421 (-3.1250, -4.1565) | 50.9997 (138.2272, 1.8117) | 32.9016 (80.268, 0) | 132.8978 (325.4725, 0.0000) | -3.5515 (-3.0126, -4.0875) |
| Greenland | 4.3449 (11.6201, 0.0215) | 2.6753 (7.206, 0.1024) | 4.8798 (12.8951, 0.1886) | -4.7181 (-4.2370, -5.1969) | 116.0653 (312.7883, 0.5256) | 64.7042 (173.874, 2.4541) | 98.3137 (262.2077, 3.7354) | -4.7025 (-4.2477, -5.1551) |
| Guam | 10.5228 (28.7322, 0) | 27.5969 (42.7733, 13.2402) | 12.9406 (20.0802, 6.2077) | -0.4187 (0.3833, -1.2144) | 284.7311 (773.9238, 0) | 733.9929 (1148.572, 353.2613) | 359.3365 (562.9593, 172.4891) | 0.4713 (1.1902, -0.2426) |
| Monaco | 12.9174 (25.9773, 2.0563) | 7.7418 (12.3245, 3.7928) | 6.3894 (10.2486, 3.1465) | -2.8518 (-2.0243, -3.6724) | 207.7719 (412.6419, 32.9129) | 117.3364 (186.605, 58.8833) | 115.1081 (183.6456, 57.7978) | -2.8583 (-2.0222, -3.6873) |
| Nauru | 1.1005 (4.0198, 0) | 1.404 (3.1515, 0.179) | 27.3783 (60.1100, 3.5851) | -1.2202 (0.1592, -2.5805) | 35.3775 (128.8845, 0) | 43.7842 (99.0596, 5.4655) | 671.5340 (1504.5798, 85.9435) | -1.2563 (0.1560, -2.6486) |
| Niue | 1.6801 (2.678, 0.6563) | 0.3632 (0.7954, 0.0349) | 17.7182 (38.8345, 1.7160) | -5.6707 (-5.1250, -6.2132) | 34.6012 (56.5876, 13.3068) | 8.0629 (17.9251, 0.7579) | 382.6371 (850.9881, 36.0419) | -5.7540 (-5.2025, -6.3023) |
| Northern Mariana Islands | 2.6931 (7.2402, 0) | 7.9845 (12.7336, 3.8096) | 19.2017 (30.7486, 9.1469) | 0.1184 (0.7631, -0.5223) | 92.113 (249.9241, 0) | 218.3547 (348.8962, 103.8372) | 420.5273 (668.7584, 200.1078) | 0.1044 (0.7569, -0.5440) |
| Palau | 1.7443 (4.7282, 0.0072) | 3.1974 (6.6915, 0.3545) | 17.9166 (37.5316, 1.9394) | 0.1114 (0.9752, -0.7450) | 47.9696 (130.9518, 0.1892) | 89.7878 (189.9114, 10.0466) | 405.2968 (853.5952, 44.6570) | 0.0144 (0.8653, -0.8294) |
| Puerto Rico | 282.7065 (910.6037, 0.0056) | 217.9958 (411.3397, 56.0638) | 2.5804 (4.8326, 0.6632) | -4.2621 (-3.7729, -4.7488) | 5520.7938 (17611.9775, 0.103) | 3857.6494 (7097.6664, 990.5895) | 55.2893 (101.9902, 14.0423) | -3.8824 (-3.3741, -4.3881) |
| Saint Kitts and Nevis | 14.0493 (27.36, 3.3456) | 5.4452 (9.5988, 1.9582) | 10.0155 (17.3700, 3.6034) | -4.2427 (-3.7568, -4.7263) | 278.4267 (545.3816, 65.8425) | 120.8239 (214.4422, 42.9331) | 186.9923 (328.6249, 67.2579) | -4.5227 (-3.9844, -5.0580) |
| San Marino | 6.9767 (12.2046, 2.584) | 3.6405 (5.9893, 1.8503) | 3.5037 (5.8211, 1.7397) | -5.0508 (-4.4918, -5.6066) | 114.3909 (199.4476, 43.2502) | 55.7468 (90.6312, 28.5834) | 66.5349 (108.3202, 33.2279) | -4.8177 (-4.3168, -5.3160) |
| Tokelau | 0.2123 (0.7135, 0.0104) | 0.1586 (0.3954, 0.0013) | 10.7738 (26.8658, 0.0909) | -2.1975 (-1.8673, -2.5265) | 4.904 (16.5921, 0.233) | 3.4665 (8.7586, 0.0293) | 237.0068 (599.8933, 2.0018) | -2.2062 (-1.8720, -2.5393) |
| Tuvalu | 10.0264 (12.3939, 7.5226) | 3.6491 (5.7169, 2.1242) | 40.1543 (62.6920, 23.5145) | -5.2258 (-5.0766, -5.3748) | 280.7879 (350.5263, 208.9816) | 95.7439 (150.5065, 54.8191) | 922.8931 (1445.7003, 535.0659) | -5.2502 (-5.1049, -5.3953) |
| United States Virgin Islands | 10.8979 (28.8801, 0.2462) | 12.376 (20.4977, 5.5121) | 7.2552 (11.9763, 3.2343) | -2.6107 (-2.2571, -2.9630) | 253.731 (672.0657, 5.6607) | 234.0643 (386.0414, 103.6296) | 139.3734 (229.2894, 61.4318) | -2.6086 (-2.2307, -2.9851) |
| South Sudan | 2529.1684 (3296.8567, 1855.5284) | 3162.2783 (4346.7848, 2274.4243) | 101.7101 (135.9572, 75.6316) | -0.5384 (-0.3104, -0.7659) | 63216.925 (82481.5401, 46663.8808) | 85559.1622 (117429.6878, 61007.2746) | 2217.1076 (3009.7320, 1604.1855) | -0.5928 (-0.3334, -0.8515) |
| Sudan | 19894.2654 (24650.7323, 15795.7084) | 25038.9426 (33720.7179, 18520.379) | 152.4323 (201.5528, 114.1875) | -1.7485 (-1.6800, -1.8170) | 509761.09 (641686.2413, 401495.0329) | 629968.0076 (863962.6713, 455425.3689) | 3137.8653 (4240.7251, 2307.9984) | -1.9316 (-1.8575, -2.0058) |
